# Comparison of Four Protocols for In Vitro Differentiation of Human Embryonic Stem Cells into Trophoblast Lineages by BMP4 and Dual Inhibition of Activin/Nodal and FGF2 Signaling

**DOI:** 10.1007/s43032-023-01334-5

**Published:** 2023-09-01

**Authors:** Zahra Anvar, Imen Chakchouk, Momal Sharif, Sangeetha Mahadevan, Li Su, Swathi Anikar, Fatemeh Alavi Naini, Alloysius Budi Utama, Ignatia B. Van den Veyver

**Affiliations:** 1 Department of Obstetrics and Gynecology, Baylor College of Medicine, Houston, TX USA; 2 Duncan Neurological Research Institute, Texas Children’s Hospital, 1250 Moursund Street, Room 1025.14, Houston, TX 77030 USA; 3 Department of Pediatrics, Baylor College of Medicine, Houston, TX USA; 4 Shared Equipment Authority, Rice University, Houston, TX USA; 5 Department of Molecular and Human Genetics, Baylor College of Medicine, Houston, TX USA

**Keywords:** Human embryonic stem cells, Differentiation, Trophoblast lineage, Syncytiotrophoblast, extravillous trophoblast

## Abstract

Human embryonic stem cells (hESCs) cultured in media containing bone morphogenic protein 4 (BMP4; B) differentiate into trophoblast-like cells. Supplementing media with inhibitors of activin/nodal signaling (A83–01) and of fibroblast growth factor 2 (PD173074) suppresses mesoderm and endoderm formation and improves specification of trophoblast-like lineages, but with variable effectiveness. We compared differentiation in four BMP4-containing media: mTeSR1-BMP4 only, mTeSR1-BAP, basal medium with BAP (basal-BAP), and a newly defined medium, E7-BAP. These media variably drive early differentiation towards trophoblast-like lineages with upregulation of early trophoblast markers CDX2 and KRT7 and downregulation of pluripotency markers (OCT4 and NANOG). As expected, based on differences between media in FGF2 and its inhibitors, downregulation of mesendoderm marker EOMES was variable between media. By day 7, only hESCs grown in E7-BAP or basal-BAP expressed HLA-G protein, indicating the presence of cells with extravillous trophoblast characteristics. Expression of HLA-G and other differentiation markers (hCG, KRT7, and GCM1) was highest in basal-BAP, suggesting a faster differentiation in this medium, but those cultures were more inhomogeneous and still expressed some endodermal and pluripotency markers. In E7-BAP, HLA-G expression increased later and was lower. There was also a low but maintained expression of some C19MC miRNAs, with more CpG hypomethylation of the *ELF5* promoter, suggesting that E7-BAP cultures differentiate slower along the trophoblast lineage. We conclude that while all protocols drive differentiation into trophoblast lineages with varying efficiency, they have advantages and disadvantages that must be considered when selecting a protocol for specific experiments.

**Supplementary Information:**

The online version contains supplementary material available at 10.1007/s43032-023-01334-5.

## Introduction

Normal development and function of the placenta is essential for a healthy pregnancy. Dysfunction of the earliest stages of trophoblast differentiation and implantation can cause common pregnancy complications, including preeclampsia, fetal growth restriction, low birth weight, early pregnancy loss, stillbirth, and preterm birth [1–3]. The human blastocyst implants into the uterine luminal epithelium at 6–9 days post fertilization. It consists of the inner cell mass (ICM), which gives rise to the embryo, and the trophectoderm (TE), which gives rise to the different types of trophoblast cells. In vitro, TE gives rise to trophoblast stem (TS) cells. TSC can also be derived from first-trimester villous cytotrophoblast (CTB). After implantation, cells of the polar TE invade through the endometrium and later the decidual glands, and fuse into the multinucleated primitive syncytium (PS) which then acquires lacunae [3–5]. The CTB cells adjacent to the ICM then form projections through the PS that merge to surround the PS as a cytotrophoblastic shell. These projecting CTB become the primitive villi containing villous cytotrophoblast (VCT) covered by a layer of fused CTB, the syncytiotrophoblast (STB). The STB is responsible for gas and nutrient exchange and secretion of human chorionic gonadotropin (hCG) and placental lactogen. These villi branch and proliferate further, then acquire a mesenchymal core (secondary villi), and next fetal capillaries (tertiary villi). Cells from the cytotrophoblastic shell further invade and become extravillous trophoblast (EVT) in anchoring villi. The EVT differentiates into two subtypes, interstitial EVT (iEVT) which invades the myometrium near the spiral arteries, and endovascular EVT (eEVT) which invades inside the spiral arteries. Remodeling of the maternal spiral arteries by EVTs is critical for optimal blood flow to the fetus [3–5].

The different stages of trophoblast development and subtypes of trophoblast cells are marked by the expression of different genes, but the processes, molecular pathways, and epigenetic modifications that control them are still being unraveled. This is due to ethical limitations and technical difficulties with accessing human trophoblast tissues at early developmental stages and to differences in placentation between humans and commonly used rodent models. One approach used to overcome these limitations is the in vitro differentiation of human embryonic stem cells (hESCs) into trophoblast lineages [6, 7]. Human ESCs are derived from the ICM of blastula-stage preimplantation embryos. Under appropriate in vitro culture conditions, they can be sustained in a pluripotent state and undergo prolonged self-renewal, but they are primed to differentiate into all three germ layers and into extraembryonic tissues. After the first report in 2002 [6], several studies have demonstrated that hESCs can differentiate into trophoblast cells when exposed to bone morphogenetic protein 4 (BMP4) [6, 8–14]. These and other similar studies differed in the selection of the specific hESC lines, the type of culture media used, the concentration of BMP4, treatment times, and frequency of media changes.

BMPs are signaling molecules belonging to the transforming growth factor beta (TGF-β) superfamily of proteins [15]. The outcome of BMP signaling is context-dependent in terms of conversion to extraembryonic lineage versus mesendoderm, which is the source of endoderm and mesoderm [16]. It has been reported that in some conditions, BMP4 preferentially drives the formation of mesoderm rather than trophoblast [17]. Removal of FGF2, a factor required for maintenance of hESC, promotes BMP4-driven trophoblast differentiation over other lineages. Furthermore, hESCs differentiate more efficiently into trophoblast-like lineages upon suppression of mesendoderm lineage differentiation when exposed to inhibitors of both fibroblast growth factor 2 (FGF2) and the activin/nodal pathway [18, 19].

Assays used to demonstrate trophoblast differentiation include downregulation of pluripotency markers octamer-binding transcription factor 4 (OCT4), SRY (sex determining region Y)-box 2 (SOX2), and Nanog homeobox (NANOG) and upregulation of trophoblast lineage markers [3–6]. Several markers can be used to assess trophoblast differentiation. Keratin7 (KRT7), major histocompatibility complex, class I, G (HLA-G), and human chorionic gonadotropin (hCG). HCG is expressed from STBs and HLA-G is uniquely expressed by EVTs [9, 20, 21]. Caudal type homeobox 2 (CDX2) which represses Oct4 in mouse hESCs [22] is the master regulator of trophoblast development in mice. Although CDX2 is highly expressed in early trophectoderm of human embryos, its role in human trophoblast development is less clear [3, 10]. The GATA binding protein 2 (GATA2), GATA binding protein 3 (GATA3), transcription factor AP-2 Alpha (TFAP2A), and transcription factor AP-2 Gamma (TFAP2C) network, with GATA3 as its pioneer factor, regulates the onset of TE differentiation [23]. The glial cells missing transcription factor 1 (GCM1) are involved in cell–cell fusion and subsequently STB formation [24]. Eomesodermin homolog (Eomes) is a marker required for the maintenance of TSC fate in mice. However, in humans, EOMES is not expressed in trophoblast and is essential for mesoderm formation [25].

Epigenetic signatures of trophoblast can also be analyzed to assess the differentiation of hESC into trophoblast lineages. One example is methylation at the promoter of *ELF5*, the gene encoding E74-like ETS Transcription Factor 5 [26]. *ELF5* is expressed throughout the cytotrophoblast in humans and the interaction of ELF5 with CDX2 is thought to be key for human trophoblast stem cell function [26]. In early human placenta, the *ELF5* promoter is hypomethylated and *ELF5* is highly expressed. In contrast, in undifferentiated hESC and hESC-derived trophoblast cells, the *ELF5* promoter is hypermethylated with associated downregulated *ELF5* expression. Upon in vitro differentiation, some studies have found continued methylation and lack of expression of ELF5, while others have seen upregulation of ELF5 expression [12, 26, 27]. The observation that, in contrast to primary trophoblasts, the *ELF5* promoter was only partially hypomethylated in hESC-derived trophoblast-like cells [20] has led to the suggestion that hESCs are an imperfect model of trophoblast development because they are epigenetically restricted from accessing the trophoblast fate due to hypermethylation at *ELF5* [7]. Alternatively, modifications to culture conditions may help drive more faithful in vitro trophoblast differentiation. Another marker characteristic of the primary trophoblast is the high expression of miRNAs from the maternally imprinted chromosome 19 miRNA cluster (C19MC) [20, 28]. These miRNAs are also expressed in hESC but have been found to decrease expression upon differentiation of hESC into trophoblast lineages [20].

These data support the importance of further experiments to test variations in trophoblast derivation methods, including the type of culture media used, the choice, duration, and dose of added differentiation factors, cell culture passage numbers, and the time course of differentiation. All those can impact the fidelity of in vitro trophoblast derivation from hESC.

In the present study, we compare four media for in vitro trophoblast differentiation from hESCs, including a new medium, E7-BAP, which is derived from the E8 pluripotency medium [29] but lacks FGF2 and contains inhibitors of TGF-β/activin/nodal signaling and FGF2. To evaluate the hESC differentiation characteristics in these media, we analyzed morphological parameters, expression of various trophoblast markers over time, and methylation of the *ELF5* promoter.

## Materials and Methods

### Cell Culture and Differentiation

**Fig. 1:**
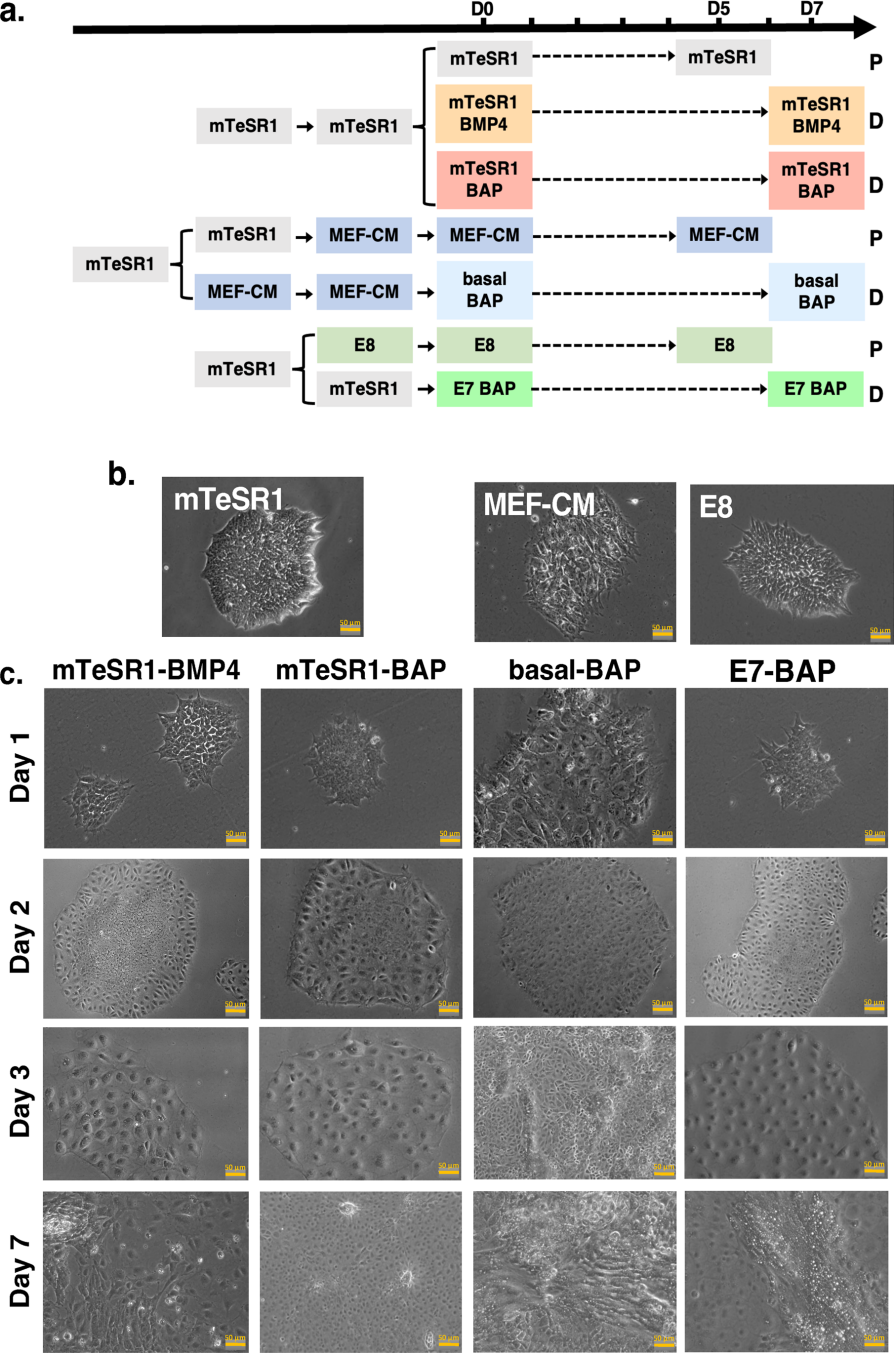
Human embryonic stem cell (hESC) differentiation protocols and colony morphology. **a** Schematic of culture conditions and timeline of the differentiation protocol. The pluripotency media (P), mTeSR1, MEF-CM, and E8, are color-coded to match the related differentiation media (D), mTeSR1-BMP4, mTeSR1-BAP, basal-BAP, and E7-BAP. The bar on top shows the timeline in days (D0-D7). **b** Phase contrast images of colony morphology of hESCs cultured in pluripotency media. **c** Phase contrast images of hESC colony morphology of cells cultured in differentiation media from day 1 to day 7. Scale bars on images are 50 μm

We obtained H9 hESCs from the Baylor College of Medicine (BCM) Stem Cells and Regenerative Medicine Center (StaR) stem cell core with appropriate WiCell use approval. Cells were at passage 33 and maintained in mTeSR1 standard stem cell medium (STEMCELL Technologies) in six-well plates coated with Matrigel (Corning #354277) before initiating the differentiation experiment. Cells were then cultured for up to seven days in four trophoblast differentiation media. In parallel, pluripotency controls were cultured for 5 days in the three corresponding pluripotency media (Fig. 1a). All media were stored at − 20 °C; thawed media were stored at 4 °C and used within two weeks. The first pluripotency medium is the mTeSR1 medium (Stemcell Technologies, Canada). The second pluripotency medium, mouse embryonic fibroblast (MEF)-conditioned medium (MEF-CM), is composed of basal medium, which is Dulbecco’s Modified Eagle Medium Nutrient Mixture F-12 (DMEM/F12; ThermoFisher Scientific, USA) with 20% Knock-Out Serum Replacement (KOSR) (Thermo Fisher Scientific, USA), 1 mM GlutaMAX (Thermo Fisher Scientific, USA), 1% nonessential amino acids (Thermo Fisher Scientific, USA) and 0.1 mM 2-mercaptoethanol (MilliporeSigma, USA)], supplemented with 4 ng/mL FGF2. This medium was first conditioned by a monolayer of γ-irradiated (8000 cGy) MEF feeder cells. MEF-CM was obtained by culturing MEFs at a density of 5 × 10^5^ cells/cm^2^ in this medium for 24 h. The conditioned media was collected and centrifuged at 3000 rpm for 10 min to remove cell debris and stored at − 80 °C until use. The third pluripotency medium, E8 [29], is DMEM/F12 with HEPES and l-glutamine (MilliporeSigma, USA), penicillin–streptomycin (Thermofisher Scientific, USA), l-ascorbic acid-2-phosphate (64 µg/mL) (MilliporeSigma, USA), sodium selenite (14 ng/mL) (MilliporeSigma, USA), insulin (20 µg/mL) (Thermo Fisher Scientific, USA), transferrin (10.7 µg/mL) (MilliporeSigma, USA), TGF-β1 (2 ng/mL) (PeproTech, USA), and FGF2 (100 ng/mL) (PeproTech, USA).

We used four different differentiation media. The first is mTeSR1-BMP4, which is mTeSR1 medium supplemented with 10 ng/mL BMP4 (PeproTech, USA). The second is mTeSR1-BAP, which is mTeSR1 medium supplemented with 10 ng/mL BMP4, 1 μM of the TGF-β type I/activin/nodal receptor (ALK4/5/7) inhibitor A83–01 (APExBIO, USA), and 0.1 μM of the FGF2 signaling inhibitor PD173074 (Selleck Chemicals, USA). The inhibitors were added to mTeSR1 to counteract the FGF2 and TGF-β in mTeSR1 and promote more effective differentiation. The third is basal-BAP, which is a basal medium supplemented with 10 ng/mL BMP4, 1 μM A83–01, and 0.1 μM PD173074. We also developed a fourth medium, which we named E7-BAP, that contains all components of the E8 pluripotency medium, except for FGF2, and is supplemented with 10 ng/mL BMP4, 1 μM A83-01, and 0.1 μM PD173074.

To drive H9 hESC cultures towards trophoblast lineage differentiation, we followed a previously described protocol [18] with some modifications (Fig. 1a). Briefly, H9 hESC colonies were seeded in mTeSR1 media and passaged to a confluence of 0.12 × 10^5^ cells/cm^2^. The following day, the media were changed to mTeSR1-BMP4, mTeSR1-BAP, or E7-BAP, for hESCs differentiated in these three media (this is considered day 0). For hESCs differentiated in basal-BAP, the mTeSR1 medium was first replaced by MEF-CM for 24 h, after which the media were exchanged to basal-BAP. Cells were grown in all four differentiation media for up to 7 days and the media were changed daily. To assess differences in efficiency and timing of differentiation, standard time points (described below) were used to assess markers of differentiation between the different cultures. Control pluripotent hESC cultures were grown in the corresponding pluripotency media, mTeSR1, MEF-CM, and E8, for up to 5 days. To mitigate the effect of first passage stress, E8 has an extra passage before d0 of the differentiation protocol. Cells were harvested for the different analyses at the time points indicated below. All cultures were done in triplicate.

### Quantitative Reverse Transcription PCR (qRT-PCR)

RNA was isolated from cell pellets frozen at − 80 °C using the miRNeasy Micro Kit (QIAGEN). RNA concentration was determined using NanoDrop UV–Vis Spectrophotometer. RNA from day 5 was used for cultures in pluripotency media and RNA from days 3, 5, and 7 was used for cultures in differentiation media. RNA from three independent cultures was analyzed for each time point. To quantify the mRNA of the eight analyzed genes, 500 ng of RNA was reverse transcribed into cDNA using the qScript cDNA SuperMix (Quanta Biosciences #95,048) and primers listed in Supplementary Table S1. To quantify the expression of miRNAs from the C19MC cluster, we applied a previously published method [20] with minor modifications. Twenty nanograms of RNA was converted into cDNA using TaqMan™ MicroRNA Reverse Transcription Kit (Applied Biosystems #4,366,596) with previously described RT primers (Supplementary Table S2) [20]. qRT-PCR quantification of reverse-transcribed cDNAs was done with PerfeCTa® SYBR® Green FastMix ROX (Quanta Biosciences #95,073) and analysis using the ΔΔCt method. Amplification of *ACTA* mRNA was used for normalizing mRNA expression and amplification of *miR-103a* was used for normalizing miRNA expression.

### Immunofluorescence Staining

For immunofluorescence (IF), hESCs from two independent cultures grown for 3 days and 7 days in differentiation media were fixed in 4% paraformaldehyde and permeabilized in PBST (phosphate buffered saline with 0.1% Tween-20). Then cells were blocked in 5% normal goat serum (Life Technologies, Cat. # 50062Z) in PBST at room temperature for 1 h. Primary antibodies were incubated at 4 °C overnight. Secondary antibodies were incubated with NucBlue Fixed Cell ReadyProbes Reagent (Thermo Fisher Scientific, Cat #R37606) at room temperature in the dark for 45–60 min [30]. IF experiments were performed on cells from two independent rounds of differentiation and control pluripotency cultures. Details on antibody dilutions and diluents used for each primary antibody are summarized in Supplementary Table S3.

All immunofluorescence-stained cultures were prepared as previously described [31]. Images were taken with a Nikon A1 Rsi confocal microscope with channels set at 1 airy unit (AU). The image settings were taken from the brightest coverslip within the same set of detected proteins to fill the dynamic range of the histogram of the protein of interest staining without oversaturation. These settings were then applied to the rest of the coverslip for the same set of detected proteins.

### Western Blot Analysis

Protein lysates were prepared as previously described from three independent cultures each on day 5 for pluripotency media and on day 7 for differentiation media [32]. Ten micrograms of protein from whole-cell lysates were denatured at 100 °C for 10 min in 10X Bolt Sample Reducing Agent and 4X Bolt LDS Sample Buffer (Life Technologies). The denatured proteins were run on a Bolt™ 4–12% Bis–Tris Plus Gels (Life Technologies, Cat. #NW04120BOX) and then transferred to a nitrocellulose membrane (GE Healthcare, Cat. #10,600,044). The transferred membrane was blocked in 5% blotting grade blocker (BIO-RAD, Cat. #170–6404) or bovine serum albumin (BSA) (RPI, Cat. #A30075-100.0) in PBST depending on the used primary antibody. The blots were incubated with primary antibodies diluted with 5% BSA or 5% blotting grade blocker overnight [31]. Anti-goat secondary antibodies (Santa Cruz Biotechnology, sc-2020) and anti-mouse and anti-rabbit secondary antibodies (Cell signaling Technologies, 7076S, and 7074S) were used for detection with Supersignal West Pico Chemiluminescent Substrate (Thermo Scientific, 34,077) or Supersignal West Femto Chemiluminescent Substrate (Thermo Scientific, Cat. #34,095) as described [31]. An antibody to alpha-Actin (ACTA1; Developmental Studies Hybridoma Bank, clone JLA20) was used as a loading control for normalizing protein expression quantification. Blot images from two independent rounds of differentiation (*n* = 3) were quantified by densitometry using Image J software.

### Methylation Analysis of the ELF5 Promoter by Bisulfite Sequencing of Individual Clones

Genomic DNA extracted from each sample was bisulfite-converted using the EZ methylation kit (ZYMO RESEARCH #D5002), according to the manufacturer’s protocol. Ten percent of the converted DNA was then used for the amplification of the − 432- to − 3-bp region upstream of the *ELF5* start site via nested PCR using previously published primers [20]. Amplicons were inserted into the pGEM-T Easy Vector System I (Promega #A1360) and DH5 $\propto$ competent cells were transformed with 2 $\upmu$ l of each ligation reaction. Ampicillin-resistant white clones were picked and were Sanger sequenced.

### Statistical Analysis

Three-way ANOVA was used for the analysis of the data from the three replicate differentiation rounds. We combined data from three rounds since the differentiation round factor did not affect the measurement of more than half of the target genes in both western blot and qRT-PCR experiments (*p* > 0.05). *p*-values were calculated using one-way ANOVA, with post hoc Tukey’s HSD test. Datasets were visualized in R with the ggpubr package. (https://github.com/kassambara/ggpubr). For qRT-PCR experiments, data were presented as mean ± standard error of the mean (s.e.m.). *p*-values were calculated using one-way ANOVA, with Dunnett’s multiple comparison test. Statistical analysis and data visualization were generated in GraphPad Prism (version 8). Where applicable, exact numbers of biological and technical replicates (*n*) for each experiment were reported in the corresponding figure legends.

## Results

### Morphology of the Human Embryonic Stem Cell Colonies

All pluripotent hESCs cultures were passaged in mTeSR1 before initiating the differentiation protocol. To obtain pluripotent control cultures for the related differentiation media (Fig. 1a), H9 hESCs were either maintained in mTeSR1 medium, or mTeSR1 was exchanged for MEF-CM and E8 pluripotency media for 5 days (Fig. 1b). Differentiation toward trophoblast lineages (Fig. 1a) was induced by adding either BMP4 alone or BAP to mTeSR1 medium, BAP to basal medium after pre-culturing hESCs for 24 h in MEF-CM, or BAP to E7 medium (which is E8 medium without FGF2), all for up to 7 days. After at least 2 days, the appearance of flattened and enlarged cells at the outside of colonies that seemed to progress towards the center, with undifferentiated cells concentrated in the center of the colony, indicated the onset of differentiation in all differentiation media (Fig. 1c). These flattened cells appeared earlier and were more uniform in mTeSR1-BMP4 and E7-BAP. Although colonies in basal-BAP appeared to be growing faster, they contained more cells with mixed morphology. By day 3, mTeSR1-BMP4, mTeSR1-BAP, and E7-BAP cultures contained colonies with large cuboidal cells, some of which appeared to have two or more nuclei. In contrast, colonies in basal-BAP continued to grow faster but continued to have cells with different morphologies, including more spindle-shaped cells. By day 7 denser areas with sheets of cells were visible in all cultures, but were less prominent in mTeSR1-BAP and more disorganized with more rapid growth in basal-BAP. In addition, cytoplasmic vesicles formed in basal-BAP cultures from day 3 onwards, and were seen in all cultures by day 7, but most prominently in basal-BAP and E7-BAP. Additional images of colony morphology of hESCs cultured on different days are shown in the online Supplementary Fig. S1.

### Trophoblast Formation by BMP4-Mediated Silencing of Pluripotency Markers and CDX2 Regulatory Switch

**Fig. 2:**
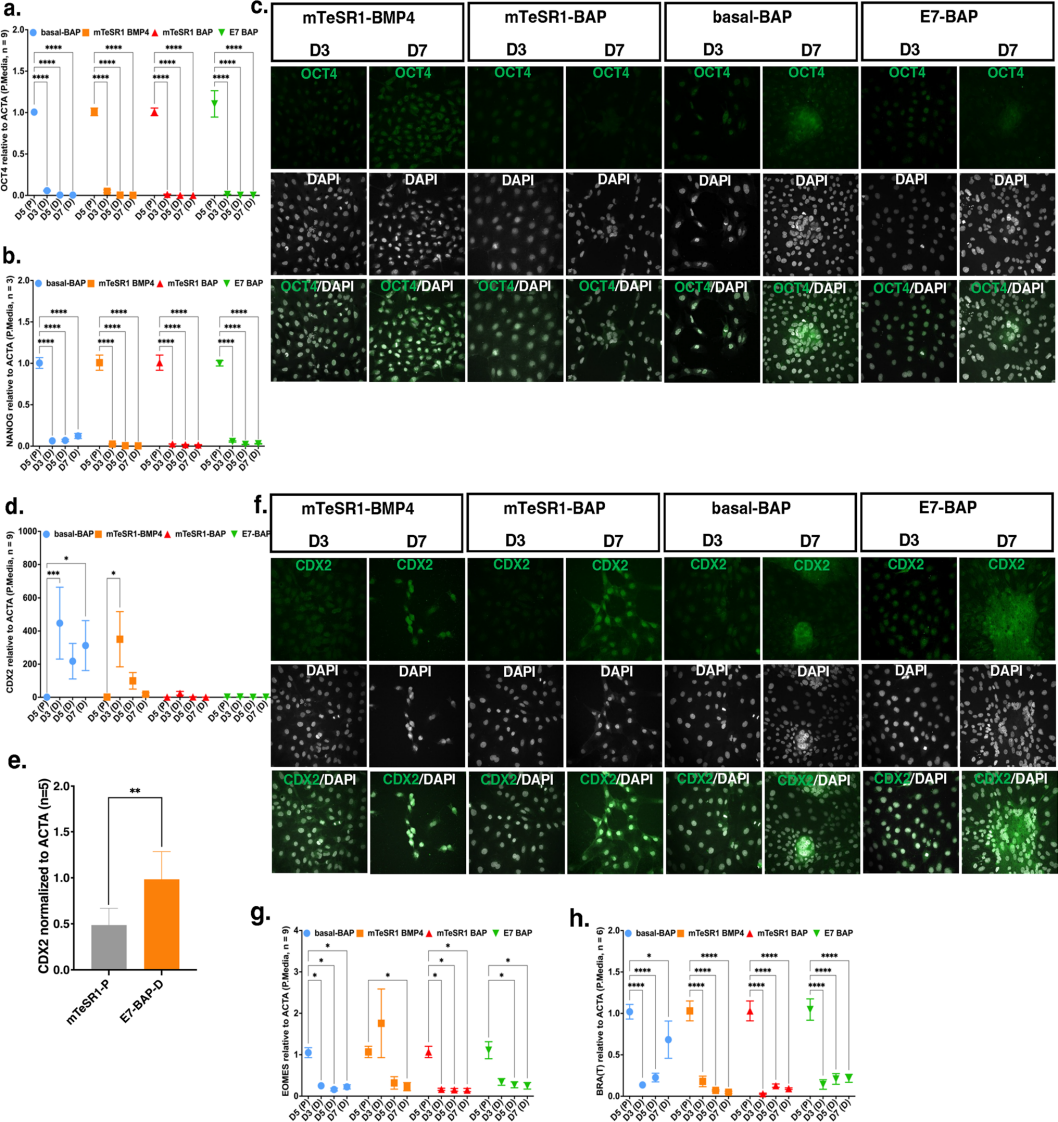
Transcript levels for *OCT4, NANOG*, *CDX2,* Brachyury (*T*), and *EOMES* and Immunofluorescence (IF) staining for OCT4 and CDX2 and western blot quantification of protein levels for CDX2. **a**, **b**, **d**, **g**, **h** Graphs of relative transcript levels of *OCT4* (**a**), *NANOG* (**b**), *CDX2* (**d**), *EOMES* (**g**), and Brachyury (*T*) (**h**) assayed by qRT-PCR and quantified using ΔΔCt normalized to *ACTA* in differentiation media (D) compared to the corresponding pluripotency media (P). D5, D3, D5, and D7 on the *X*-axis represent days in culture in each differentiation medium and matching pluripotency medium. Relative transcript levels and the number of replicates for each gene are on the *Y*-axis. Data are presented as mean $\pm$ standard error of the mean (SEM); *p < 0.05, **p < 0.01, ***p < 0.001, ****p < 0.0001. **c**, **f** Images of cultured cells stained on day 3 (D3) and day 7 (D7) for markers OCT4 (**c**) and CDX2 (**f**) (left images) and for each marker co-stained with nuclear marker DAPI (right images) in differentiation media. Each IF staining was performed in triplicate from two independent rounds of differentiation. **e** Western blot quantification of protein levels of CDX2. Lysates were collected on day 7 from cells cultured in E7-BAP and on day 5 from cells cultured in mTeSR1 as the pluripotent media; the Graph shows the relative protein levels assayed by densitometry normalized to ACTA (*Y*-axis). Number of replicates is shown on *Y*-axis (*n* = 5), **p < 0.01

On day 3 of differentiation, as expected, *OCT4* and *NANOG* transcripts were significantly lower in mRNA extracted from hESCs cultured in all four differentiation media compared to pluripotency media (*p* < 0.0001 for both *OCT4* and *NANOG*). They became undetectable by day 5 and remained absent on day 7, confirming the loss of pluripotency (Fig. 2a, b). Similarly, no nuclear OCT4 immunofluorescence staining was observed on day 3 and day 7 in all differentiation media, except for basal-BAP, where some cells showed possible nuclear staining, suggesting the remaining presence of pluripotent cells. Some cells in different cultures show very faint cytoplasmic background staining, which we interpret as non-specific background since OCT4 is a nuclear protein (Fig. 2c).

In humans, CDX2 is only detected in the trophectoderm of established blastocysts, and its expression does not repress the trophectoderm OCT4 expression [33, 34]. *CDX2* transcript levels peaked on day 3 in hESC cultured in basal-BAP (*p* < 0.001) after which they decreased but remained elevated on day 5 and day 7 (*p* < 0.05). *CDX2* transcripts also peaked on day 3 in hESCs cultured in mTeSR1-BMP4 (*p* < 0.05) but declined by day 5 to very low levels on day 7 (Fig. 2d). Western analysis for CDX2 protein expression in E7-BAP on day 7 also confirmed its higher expression in that medium on day 7 (Fig. 2e). CDX2 western blot images of five replicates between the E7-BAP as the differentiation media and mTesR1 as the pluripotency media are shown in Supplementary Fig. S2. In hESCs cultured in mTeSR1-BAP and E7-BAP, transcript levels were not changed upon differentiation compared to levels in undifferentiated cultures (Fig. 2d). Immunofluorescence staining did not detect nuclear CDX2 on day 3 in cells cultured in any of the four differentiation media (some cultures had very faint non-specific cytoplasmic staining) (Fig. 2f). On day 7, staining was variable in cells cultured in mTeSR1-BMP4 and mTeSR1-BAP, with a few colonies showing stronger nuclear staining, while in basal-BAP and E7-BAP, staining was brighter in areas of high cell density that appeared to contain multinucleated cells in some denser parts with irregular-appearing nuclei in basal-BAP medium.

### Primitive Streak and Mesendoderm Marker Expression

We assessed transcript levels for *EOMES* and Brachyury (*T*), both T-box transcription factors that are essential for the formation of endoderm and mesoderm [35]. *EOMES* transcript levels were very low on day 3 and nearly undetectable on day 5 in hESCs cultured in mTeSR1-BAP, basal-BAP, and E7-BAP (all with *p* < 0.05). In hESCs cultured in mTeSR1-BMP4 media, *EOMES* transcripts appeared higher on day 3, but this was not statistically significant. They declined to nearly undetectable levels by day 7 (*p* < 0.05) in all four differentiation media (Fig. 2g). These data indicate likely initial mesoderm induction with mTeSR1-BMP4, but effective suppression of mesoderm in all cultures that also contained inhibitors to FGF2 and activin/nodal (BAP), consistent with prior data. Compared to hESCs grown in pluripotency media, *T* transcript levels were almost undetectable by day 3 in cells grown in all four differentiation media (*p* < 0.0001). However, in basal-BAP, *T* transcript levels were again expressed at higher levels on day 7 (*p* < 0.05 compared to day 5) (Fig. 2h). This supports that basal-BAP is not effective in suppressing endodermal fate, consistent with the mixed-cell morphology in those cultures.

### Trophectoderm Four (TEtra) Elements, TFAP2B, and KRT7 Pan-trophoblast Markers

The “trophectoderm four” (TEtra) elements, TFAP2A, TFAP2C, GATA2, and GATA3 transcription factors represent a network that links exit from pluripotency by suppression of OCT4 to the emergence of early trophoblast lineage progenitors [23]. Pan-trophoblast marker cytokeratin 7 (KRT7 or CK7), is a commonly studied epithelial marker expressed by all trophoblasts but not villous stroma [20].

**Fig. 3:**
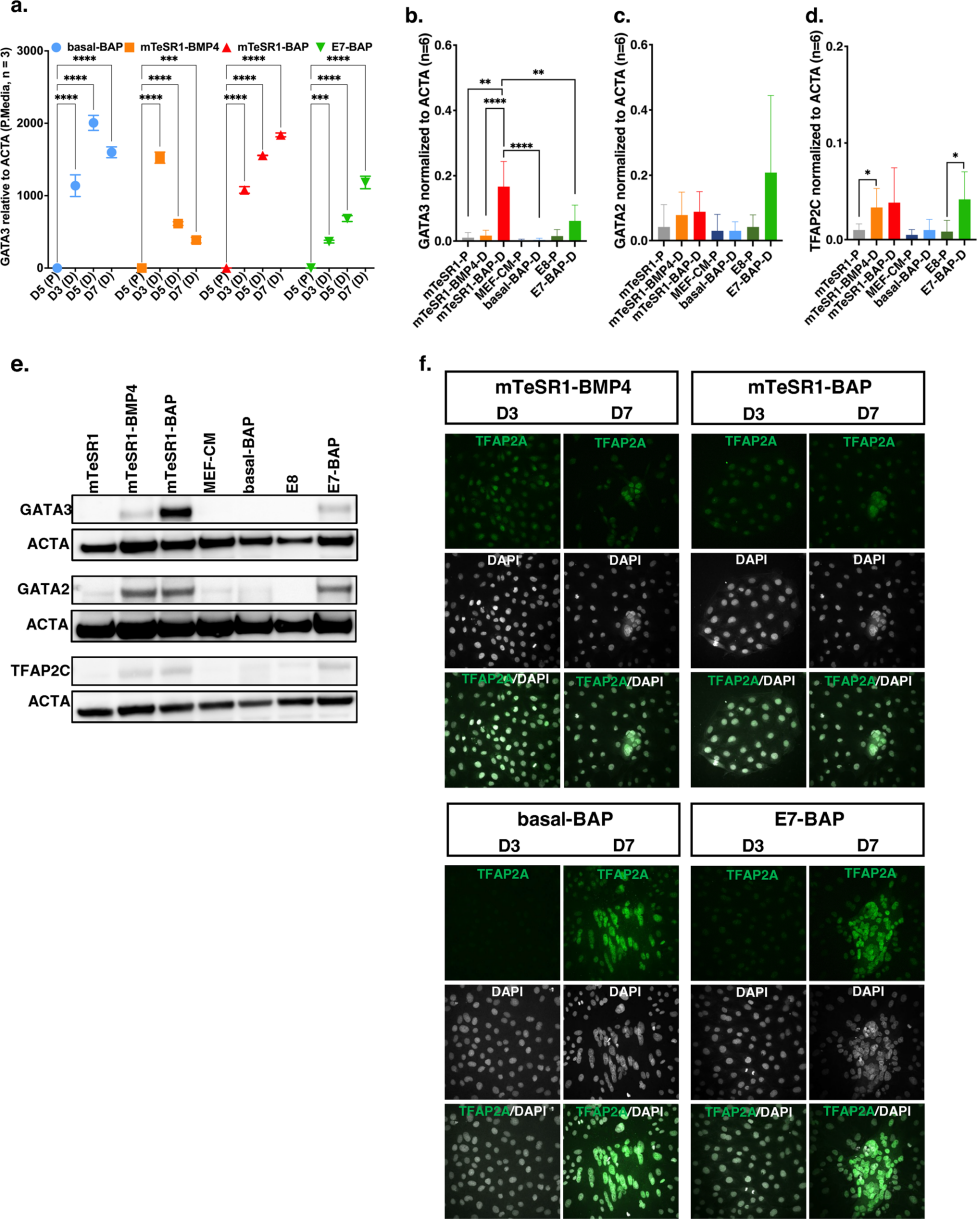
Transcript levels for *GATA3*, western blot quantification of protein levels for GATA3, GATA2, and TFAP2C and Immunofluorescence (IF) staining for TFAP2A. **a** Relative transcript levels of *GATA3* assayed by qRT-PCR and quantified using ΔΔCt normalized to *ACTA* in differentiation media (D) compared to the corresponding pluripotency media (P). D5, D3, D5, and D7 on the *X*-axis represent days in culture in each differentiation medium and matching pluripotency medium. Relative transcript levels and number of replicates are on the *Y*-axis. Data are presented as mean $\pm$ standard error of the mean (SEM); ***p < 0.001, ****p < 0.0001. **b–d** Western blot quantification of protein levels of GATA3, GATA2 and TFAP2C and corresponding western blot image (**e**). Lysates were collected on day 7 from cells cultured in differentiation media and on day 5 from cells cultured in matching pluripotent media. The graph shows the relative protein levels assayed by densitometry normalized to ACTA (*Y*-axis) in the respective media (*X*-axis). Data are obtained from two independent rounds of differentiation (*n* = 6). *p < 0.05, **p < 0.01, ***p < 0.001, ****p < 0.0001. **f** Images of cultured cells stained on day 3 (D3) and day 7 (D7) for TFAP2A (left images) and co-stained with nuclear marker DAPI (right images) in differentiation media. IF staining was performed in triplicate from two independent rounds of differentiation

Transcript levels of the Tetra pioneering factor, *GATA3* gradually increased during differentiation in mTeSR1-BAP and E7-BAP until day 7 (both with *p* < 0.001), whereas in mTeSR1-BMP4 and basal-BAP, *GATA3* transcripts levels peaked on day 3 and day 5, respectively (both *p* < 0.0001), after which they decreased again, but were still higher than in undifferentiated cultures (*p* < 0.001 and *p* < 0.0001, respectively), consistent with an early role in trophoblast differentiation and slower differentiation in mTeSR1-BAP and E7-BAP (Fig. 3a). Western analysis of lysates on day 7 showed that GATA3 protein levels were significantly higher in cultures grown in mTeSR1-BAP compared to either differentiation or pluripotency media (*p* < 0.01). More protein was also detected in E7-BAP cultures compared to E8 pluripotent cultures, but the difference did not reach significance (Fig. 3b, e; Supplementary Fig. S3). Western analysis of cell lysates obtained on day 7 in differentiation media and day 5 in pluripotency media showed that although GATA2 protein levels appeared higher after differentiation in mTeSR1-BMP4, mTeSR1-BAP, and E7-BAP, this was not significant (*p* = 0.056) and there were no differences between cultures grown in the various differentiation media (Fig. 3c, e; Supplementary Fig. S3).

TFAP2C protein levels were also assayed by western analysis, which showed that levels were significantly higher in lysates from cultures grown in mTeSR1-BMP4 (*p* < 0.05) and E7-BAP (*p* < 0.05) on day 7 of differentiation, compared to day 5 cultures in corresponding pluripotency media (Fig. 3d, e; Supplementary Fig. S2). We detected nuclear staining by immunofluorescence for TFAP2A from the third day of differentiation onward in colonies grown in all media, while on day 7, colonies were stained prominently (Fig. 3f).

**Fig. 4:**
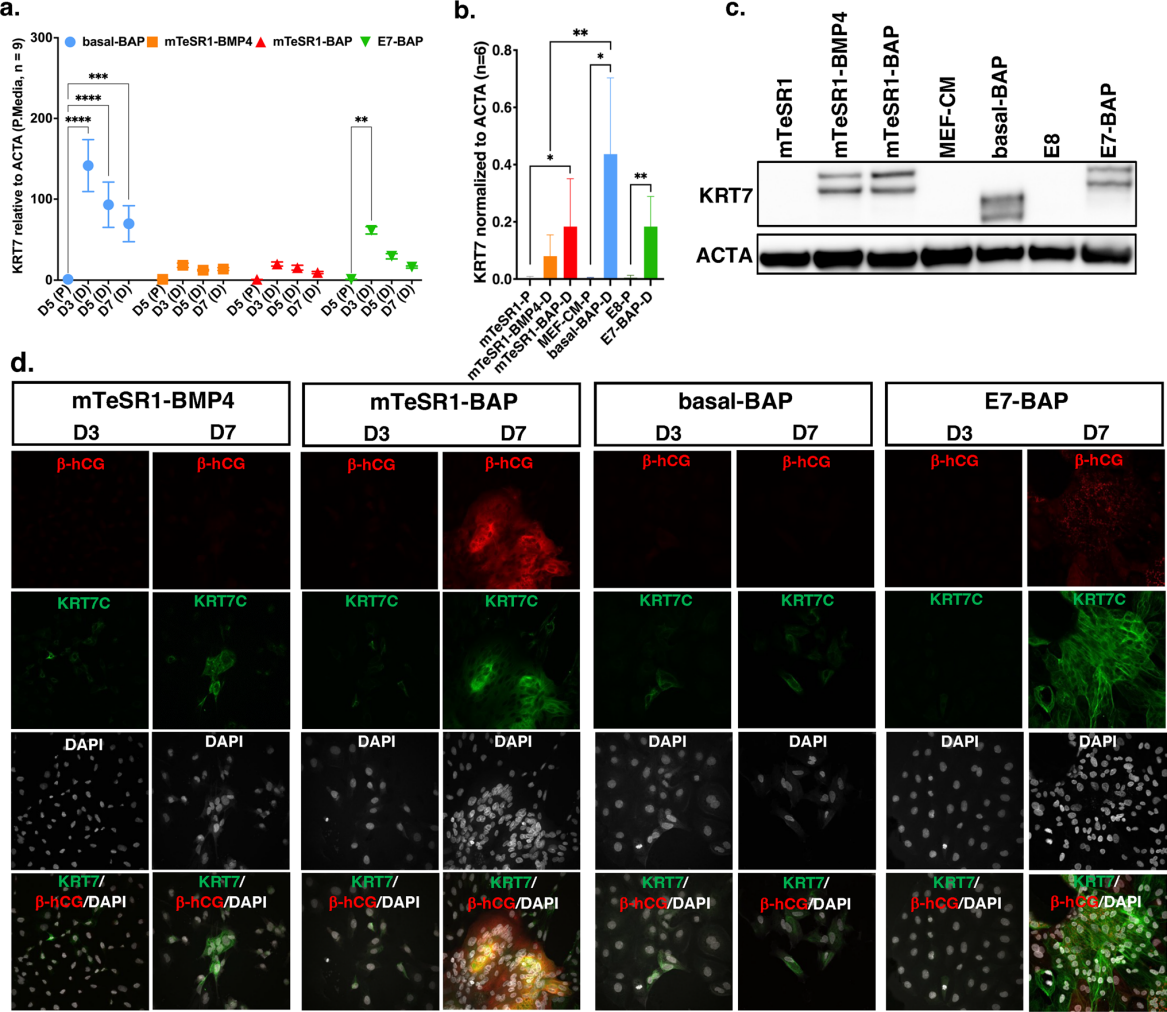
Transcript levels, and Western blot quantification of protein levels for KRT7 and Immunofluorescence (IF) staining for KIRT7 and $\beta$-hCG. **a** Relative transcript levels of *KRT7* assayed by qRT-PCR and quantified using ΔΔCt normalized to *ACTA* in differentiation media (D) compared to the corresponding pluripotency media (P). D5, D3, D5, and D7 on the *X*-axis represent days in culture in each differentiation medium and matching pluripotency medium. Relative transcript levels and number of replicates are on the *Y*-axis. Data are presented as mean $\pm$ standard error of the mean (SEM); *p < 0.05, **p < 0.01, ***p < 0.001, ****p < 0.0001. **b** Western blot quantification of protein levels of KRT7 and corresponding western blot image (**c**). Lysates were collected on day 7 from cells cultured in differentiation media and on day 5 from cells cultured in matching pluripotent media. The graph shows the relative protein levels assayed by densitometry normalized to ACTA (*Y*-axis) in the respective media (*X*-axis). Data are obtained from two independent rounds of differentiation (*n* = 6). *p < 0.05, **p < 0.01. **d** Images of cultured cells stained on day 3 (D3) and day 7 (D7) for KRT7 and $\beta$-hCG (left and middle images) and co-stained with nuclear marker DAPI (right images) in differentiation media. IF staining was performed in triplicate from two independent rounds of differentiation

We next examined transcript and protein levels for the universal trophoblast marker, cytokeratin 7 (KRT7). *KRT7* transcript levels were the highest on day 3 compared to other time points in all four differentiation media. On day 3, *KRT7* transcripts were significantly higher in both basal-BAP (*p* < 0.0001) and E7-BAP (*p* < 0.01) compared to those in corresponding pluripotency media (Fig. 4a). Western blots of lysates showed the same patterns with significantly more KRT7 protein in lysates from cells grown in basal-BAP (*p* < 0.05), E7-BAP (*p* < 0.01) and also mTeSR1-BAP (*p* < 0.05) compared to their respective pluripotent media (Fig. 4b, c; Supplementary Fig. S3). There was very weak cytoplasmic immunostaining for KRT7 on day 3 in a few cells of colonies grown in all differentiation media. On day 7, all colonies stained distinctly in all differentiation media, with more cells staining strongly in mTeSR1-BAP and E7-BAP and the latter providing the most distinct staining pattern showing large sheets of KRT7-positive cells. As expected, there was partial overlap with cells that stained with an antibody against the β subunit of hCG in cultures in mTeSR1-BAP and E7-BAP on day 7 (Fig. 4d).

### Markers of Syncytiotrophoblast and Extravillous Trophoblast Differentiation

**Fig. 5:**
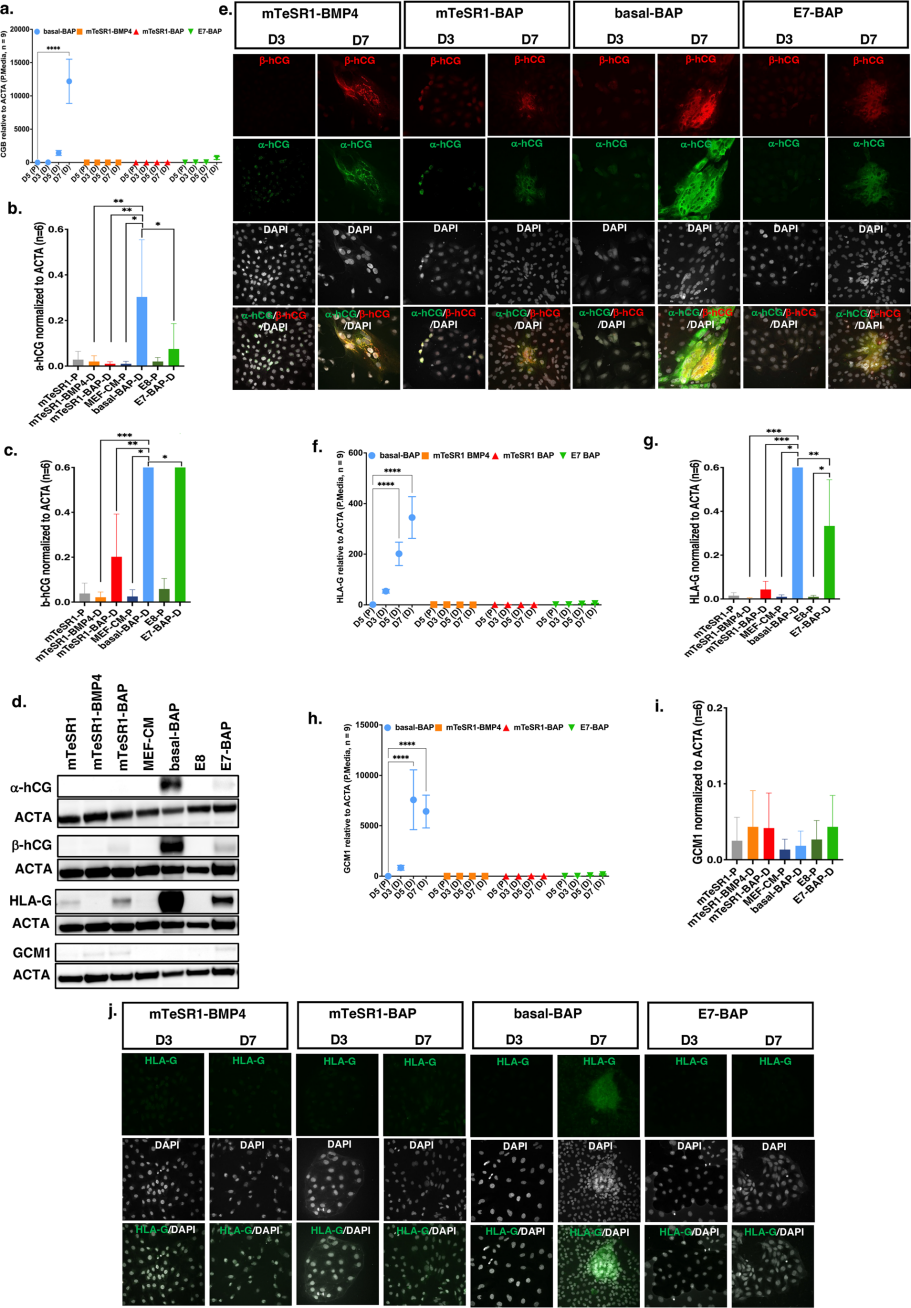
Transcript levels for *CGB*, *HLA-G*, and *GCM1*, Immunofluorescence (IF) staining for $\alpha$-hCG, $\beta$-hCG, and HLA-G and Western blot quantification of protein levels for $\alpha$-hCG, $\beta$-hCG, HLA-G, and GCM1. **a**, **f**, **h** relative transcript levels of *CGB* (**a**), *HLA-G* (**f**), and *GCM1* (**h**) assayed by qRT-PCR and quantified using ΔΔCt normalized to *ACTA* in differentiation media (D) compared to the corresponding pluripotency media (P). D5, D3, D5, and D7 on the *X*-axis represent days in culture in each differentiation medium and matching pluripotency medium. Relative transcript levels and number of replicates are on the *Y*-axis. Data are presented as mean $\pm$ standard error of the mean (SEM); ****p < 0.0001. **b**, **c**, **g**, **i** Western blot quantification of protein levels of $\alpha$-hCG (**b**), $\beta$-hCG (**c**), HLA-G (**g**), and GCM1 (**i**) and corresponding western blot image (**d**). Lysates were collected on day 7 from cells cultured in differentiation media and on day 5 from cells cultured in matching pluripotent media. Each graph shows the relative protein levels assayed by densitometry normalized to ACTA (*Y*-axis) in the respective media (*X*-axis). Data are obtained from two independent rounds of differentiation (*n* = 6). *p < 0.05, **p < 0.01, ***p < 0.001, ****p < 0.0001. **e**, **j** Images of cultured cells stained on day 3 (D3) and day 7 (D7) for $\alpha$-hCG, $\beta$-hCG (left and middle images) and co-stained with nuclear marker DAPI (right images) (**e**) and for HLA-G (**j**) in differentiation media. IF staining was performed in triplicate from two independent rounds of differentiation

The major placental glycoprotein hormone produced primarily by syncytiotrophoblasts is hCG, a heterodimer composed of a unique beta subunit (β-hCG) encoded by six possible *CGB* genes and an alpha subunit (α-hCG) shared with other heterodimeric hormones (LH, FSH, and TSH) and encoded by the *CGA* gene. We examined the expression of *CGB* using a primer pair that amplifies common regions of *CGB* transcripts by quantitative RT-PCR. *CGB* transcripts were present in RNA extracted from cells grown in basal-BAP after day 5 and with significantly elevated levels on day 7 (*p* < 0.0001). No *CGB* transcript was detected on any day in cells grown in other media (Fig. 5a). Western analysis with antibodies to either α-hCG or β-hCG protein subunits showed the highest protein levels on day 7 in cells grown in basal-BAP. There was high β-hCG (*p* < 0.05) in cells grown in E7-BAP, but no expression of α-hCG or β-hCG was detected in any other cell lysates (Fig. 5b–d; Supplementary Fig. S3). Co-immunostaining with antibodies to α-hCG and β-hCG did not reveal positive cells on day 3 of differentiation. There were distinct differences between media on day 7 with weak staining for α-hCG or β-hCG in colonies grown in mTeSR1-BMP4, only staining for α-hCG in mTeSR1-BAP, and strong overlapping staining for both in colonies grown in basal-BAP and E7-BAP (Figs. 5d and 5e). This was most prominent in denser sheet-like areas with cells that appeared multinuclear, supporting more effective differentiation towards syncytiotrophoblast in those media (Fig. 5e).

We also examined the expression of *HLA-G*, a marker that correlates with the degree of CTB invasiveness and is not expressed in the non-invasive STB of the chorionic villi [36]. Colonies cultured in basal-BAP had higher levels of *HLA-G* transcripts (Fig. 5f). Western analysis of lysates showed higher levels of HLA-G protein in basal-BAP (*p* < 0.05) and E7-BAP (*p* < 0.05) compared to corresponding pluripotency media, but no HLA-G was detected in any other differentiation media (Fig. 5d, g). Immunostaining confirmed HLA-G expression on day 7 in colonies grown in basal-BAP, but not in E7-BAP (Fig. 5j). Thus, combined hCG and HLA-G expression studies suggest that E7-BAP cultures yielded more differentiation towards hCG-expressing STB, while cells grown in basal-BAP medium achieved both STB and EVT lineage differentiation.

*GCM1* plays a role in the regulation of syncytialization of cytotrophoblast [37]. Transcript abundance of *GCM1* increased starting day 3 and reached a highly stable level by day 5 (*p* < 0.0001) that was maintained on day 7 in colonies cultured in basal-BAP, but it was not altered in other media. Although a small increase was seen on day 7 in E7-BAP, it was not significant (Fig. 5h). Western analysis of day 7 lysates detected low expression of GCM1 protein in all culture conditions with no significant difference between different culture media (Fig. 5d, i).

### The Abundance of C19MC miRNAs and ELF5 Promoter Methylation

**Fig. 6:**
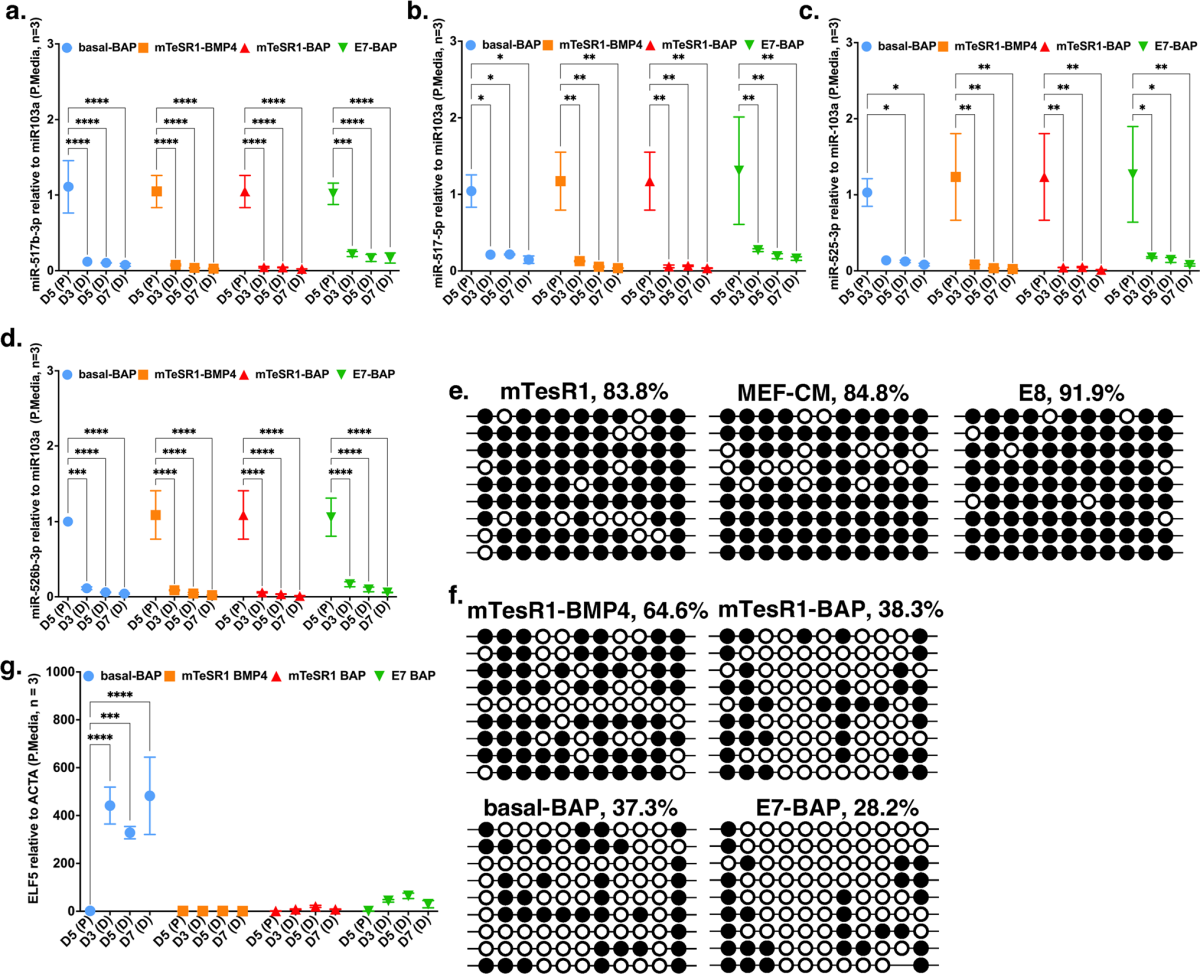
Abundance of selected miRNAs from the C19MC complex and promoter CpG methylation and transcript levels of *ELF5.* **a**–**d** Graphs of relative transcript levels of four C19MC miRNAs (**a** *miR517b-3p*; **b** *miR517b-5p*; **c** *miR525-3p*; **d** *miR526b-3p*) assayed by qRT-PCR and quantified using ΔΔCt normalized to *miR103a* in corresponding pluripotency media in hESCs grown in differentiation media (D) on days 3, 5, and 7 of differentiation, compared to hESC cultured in matching pluripotent media (P) collected on day 5. Days in culture in each differentiation medium and matching pluripotency medium are shown on the *X*-axis. Relative miRNA levels and number of replicates for each gene are on the *Y*-axis. Data are presented as mean $\pm$ standard error of the mean (SEM) and each experiment was performed in triplicate (*n* = 3). *p < 0.05, **p < 0.01, ***p < 0.001, ****p < 0.0001. **e**, **f** *ELF5* promoter methylation analysis by bisulfite sequencing of individual clones in cells cultured in pluripotency media (**e**) and differentiation media (**f**) on day 7. Each row represents an individual sequenced clone; methylated and unmethylated CpG dinucleotides are represented as open and filled circles, respectively. Percentages show the proportion of methylated to non-methylated CpG sites for each set of cloned PCR products. **g** Relative transcript levels of *ELF5* assayed by qRT-PCR and quantified using ΔΔCt normalized to *ACTA* in differentiation media (D) compared to the corresponding pluripotency media (P). D5, D3, D5, and D7 on the *X*-axis represent days in culture in each differentiation medium and matching pluripotency medium. Relative transcript levels and number of replicates are on the *Y*-axis. Data are presented as mean $\pm$ standard error of the mean (SEM); ***p < 0.001, ****p < 0.0001

Previous studies have shown that primary first-trimester trophoblast and choriocarcinoma cell lines (JEG-3, JAR) have a high abundance of C19MC miRNAs and that H9 hESCs have moderate levels of C19MC miRNAs which further decreased upon differentiation into trophoblast [20]. All four C19MC miRNAs that were examined (has-miR-517-5p, has-miR-517b-3p, has-miR-525-3p, has-miR-526b-3p) were expressed in hESCs grown in the three-pluripotency media (Fig. 6a–d). Transcript levels of all four C19MC miRNAs were lower by day 3 of differentiation in all media compared to their pluripotent media, and remained relatively low thereafter, except for levels of miR-525-3p in hESC grown in basal-BAP, which were not significantly lower until day 5 (*p* < 0.05). Levels of miR-517-5p in E7-BAP and basal-BAP and of miR-517b-3p in E7-BAP appeared slightly higher than in the other differentiation media at each time point, but this difference was not significant (*p* > 0.05).

In early human placenta, the *ELF5* promoter is hypomethylated and *ELF5* is highly expressed. It has been shown that the *ELF5* promoter in hESC is hypermethylated and *ELF5* gene expression is downregulated [20, 26]. We examined methylation of the *ELF5* promoter in hESC grown in pluripotency media and quantified how it changes upon differentiation in the four differentiation media. As expected, we found that the *ELF5* promoter is highly methylated in pluripotent hESCs in all examined pluripotency media (between 83.8 and 91.9%) (Fig. 6e). After 7 days of differentiation, methylation of the *ELF5* promoter was reduced to 64.6% in mTeSR1-BMP4, to 38.3% in mTeSR1-BAP, to 37.3% in basal-BAP, and was the lowest in E7-BAP at 28.2% (Fig. 6f). Transcript levels assessed by qRT-PCR showed high persistent levels starting on day 3 in cells grown in basal-BAP compared to transcript level in corresponding pluripotency media (*p* < 0.0001). A smaller increase that did not reach significance was observed in cells grown in E7-BAP on day 3 (Fig. 6g).

## Discussion

In this study, we evaluated three published protocols and culture conditions along with a protocol developed in our laboratory. We examined morphological changes over seven days of in vitro differentiation. We also studied various trophoblast differentiation markers by quantitative RT-PCR of expressed transcripts and by western analysis and immunofluorescence staining of expressed proteins. Finally, we assayed changes in methylation at the *ELF5* promoter and in the expression of the C19MC miRNA cluster. Our results indicate that, consistent with other data [6, 8–11, 18, 19, 38], all differentiation media can drive trophoblast lineage differentiation from the periphery towards the center of hESC colonies, but to variable degrees and at a variable pace (differences in silencing of pluripotency markers and the expression levels of certain other markers are summarized in Supplementary Table S4). For our primary analysis, we compared the marker expression analysis for the new E7-BAP differentiation protocol to its corresponding pluripotency medium, E8. To verify that the additional step required for culturing hESC in the E8 pluripotency medium (see Fig. 1a) does not influence the results, we also compared qRT-PCR marker expression data in E7-BAP to the mTeSR1 pluripotency medium and found equivalent results (Supplementary Figs. S4 and S5).

Human ESC cultured in a standard pluripotency medium have an epithelial morphology, high levels of DNA methylation, and a transcriptome resembling primed post-implantation epiblast. Therefore, they have been referred to as epiblast-type or primed cells [39]. Different reported protocols for differentiation of primed human embryonic stem cells along trophoblast lineages have shown variable results, which has led some to debate the ability of hESC to differentiate into trophoblast in these culture systems [17, 40]. After the initial report by Xu et al. that BMP4 drives trophoblast differentiation [6], approaches of BMP4-driven differentiation of hESC in defined media had variable results, with some yielding primarily mesoderm [17] or endoderm [41]. Further studies have shown that more effective BMP4-driven differentiation towards trophoblast requires suppression of FGF2 and activin/nodal signaling, but there remains variation in the details between these protocols that affect the fidelity and efficiency of differentiation towards trophoblast. This can be influenced by ambient O_2_ conditions, the source and concentration of BMP4 used, starting sizes of colonies, and initial colony density at the time of BMP4 addition [41]. In some reports, FGF2 was present [6, 12, 16, 42] and in other reports, it was excluded at the time BMP4 was added [8, 43–45]. For example, Wei and colleagues used a novel E6 medium, which lacks TGF-β and bFGF, to differentiate induced pluripotent stem cells (iPSCs) into trophoblast-like cells. They observed that the iPSC-derived trophoblast-like cells had similar gene expression profiles and displayed syncytialization and invasiveness in Matrigel [45].

We obtained effective downregulation of pluripotency factors in all differentiation media, except for a slight upregulation of *NANOG* in basal-BAP, in which cells also proliferated much faster than in other media. The mTeSR1 medium containing only BMP4 resulted in slower differentiation that started later and was associated with less effective suppression of mesodermal fate, as evidenced by *EOMES* expression on day 3, and with less effective syncytialization. This is consistent with other observations of upregulation of mesoderm and endoderm markers in this culture medium [16, 18, 19]. Colonies cultured in mTeSR1 with BMP4 or BAP for 7 days did not produce *CGB* transcripts and β-hCG was not seen by western analysis. These colonies also had variable and lower evidence of β-hCG-positive cells by immunofluorescence. In contrast, the fastest upregulation of *CGB* transcripts (by day 3) was in cells cultured in basal-BAP, followed by cells cultured in E7-BAP, both of which showed sheets of multinucleated cells with a syncytial appearance.

Basal-BAP was the only medium that also led to strong and early upregulation of *HLA-G* transcripts by day 3 and significantly elevated HLA-G protein levels by western analysis on day 7. Culturing in E7-BAP resulted in lower but still significant upregulation of *HLA-G* transcripts from day 5 onwards and HLA-G protein was identified by western analysis on day 7. However, the only cultures that contained cells with HLA-G by immunofluorescence labeling were basal-BAP cultures on day 7. This could suggest that only basal-BAP drives efficient differentiation into EVT lineages, and that cells that acquire EVT characteristics are rarer with differentiation in E7-BAP. This is consistent with other studies wherein FGF2 inhibition together with in vitro differentiation with BMP4 or BAP primarily drives STB differentiation and not EVT lineages [10, 11, 20, 43, 46]. Alternatively, in E7-BAP EVT lineages may appear later and possibly in a more controlled fashion, but evaluating this will require future studies with longer cultures in E7-BAP. While the strong expression of HLA-G in basal-BAP, along with the highest and earliest expression of several other markers of trophoblast differentiation (*GATA3*, *TFAP2C*, *KRT7*, *CDX2*, and *GCM1*) compared to other cultures at first may appear promising, this medium has several disadvantages that make it less optimal for experiments requiring a purer trophoblast population. Morphologically, the cultures are more mixed and disorganized, proliferate more rapidly, and re-express *NANOG* transcripts on day 7, suggesting that cells have maintained higher proliferative capacity. One limitation of our study was that we did not test for the absence or downregulation of HLA-A, HLA-B, and HLA-C expression, which are important to evaluate the efficiency and purity of trophoblast lineage differentiation [47]. Another limitation of our study is that although in our IF experiments we used validated antibodies and all experiments were performed on cells from two independent rounds of differentiation with appropriate control pluripotency cultures, we did not include a specific positive control for each marker with nuclear staining and a negative control to detect any background staining.

To further evaluate how well the differentiated cells adopted trophoblast identity, we examined two additional markers, expression of the C19MC miRNA cluster and methylation of the *ELF5* promoter [20]. High expression of C19MC miRNAs is a hallmark of first-trimester trophoblast, increases as gestation progresses, and is also implicated in the regulation of EVT invasion [20, 48, 49]. We found that all four miRNAs of the C19MC cluster we tested were downregulated by day 3 in all differentiation media, which has been observed before in hESC differentiation towards trophoblast lineages [20]. This result may reflect the incomplete differentiation on day 7 and we speculate that longer cultures may increase the C19MC miRNA expression and more advanced first-trimester trophoblast-like identity, possibly more in the E7-BAP media. This can be tested in future longer-term experiments. *ELF5* promoter methylation is considered an epigenetic modification that keeps extraembryonic and embryonic lineages separate; in the human placenta, the promoter is hypomethylated and the gene is expressed, although promoter methylation increases from the first to the third trimester of pregnancy [26]. The *ELF5* promoter is highly methylated in non-trophoblast placental villous mesenchymal cells and is hypomethylated to different degrees in EVTs and vCTBs, but these lower methylation levels are not associated with strong expression [20]. Earlier studies of in vitro differentiation showed that *ELF5* is hypermethylated and repressed in hESC and remains hypermethylated in derived trophoblast cell lines, suggesting that these cells did not exhibit the epigenetic signature of trophoblast lineages [20, 26]. We confirmed that the *ELF5* promoter was highly methylated in hESCs cultured in pluripotency media, consistent with older previous studies [20, 26, 27]. However, unlike what has been reported in some previous studies [26], but consistent with other data [12], it became hypomethylated to varying degrees in differentiation media, with E7-BAP resulting in the lowest methylation levels (Fig. 6f). This supports that these cells began to acquire epigenetic characteristics of trophoblast lineages.

In conclusion, we confirmed the ability of BAP to generate trophoblast-like cells in four examined media including a newly developed defined medium, E7-BAP, which lacks FGF2. This combination adequately suppressed mesendodermal factors and emphasizes the importance of FGF2 inhibition for preventing BMP4-mediated differentiation into mesoderm and endoderm lineages. We also found that compared to other media, differentiation in E7-BAP resulted in more hypomethylation of the ELF5 promoter, supporting an epigenetic state closer to that seen in the first-trimester trophoblast.

### Supplementary Information

Below is the link to the electronic supplementary material.

Supplementary file1 (PDF 15520 KB)

Supplementary file2 (DOCX 28 KB)

## Data Availability

Not applicable.
