# Supplementary material for: Comparison of Four Protocols for In Vitro Differentiation of Human Embryonic Stem Cells into Trophoblast Lineages by BMP4 and Dual Inhibition of Activin/Nodal and FGF2 Signaling
Source: Reprod Sci. 2023 Sep 1;31(1):173–89. doi: 10.1007/s43032-023-01334-5 (PMC10784360; doi:10.1007/s43032-023-01334-5)

Supplementary Figures S1-S5:

Anvar Z, Chakchouk I, Sharif M, Mahadevan S, Su L, Anikar S, Alivi F, Budi Utama A, Van den Veyver IB.

Comparison of four protocols for in vitro differentiation of human embryonic stem cells into trophoblast lineages by BMP4 and dual inhibition of Activin/Nodal and FGF2 signaling.

In: *Reproductive Sciences 2022*
Correspondence: Ignatia B Van den Veyver: iveyver@bcm.edu

Legends:

Supplementary Fig. S1. Additional images of human embryonic stem cell (hESC) colony morphology in pluripotency and differentiation media

Phase contrast images of hESC colony morphology of cells cultured on Day (D) 4, 6 and 7 in differentiation media. Scale bars on images are 50 um.

Supplementary Fig. S2. Western blot images of replicates of CDX2

Lysates were collected on day 7 from cells cultured in E7-BAP as differentiation media (D) and on day 5 from cells cultured in mTeSR1 as pluripotency media (P). CDX2 Protein levels were assayed and quantified by densitometry of each protein normalized to ACTA. Western blots show five replicates for the experiment. Densitometry values were used in graph in Fig 2e.

Supplementary Fig. S3. Western blot images of replicate rounds of differentiation

Lysates were collected on day 7 from cells cultured in differentiation media (D) and on day 5 from cells cultured in matching pluripotent media (P). Markers tested were GATA3, GATA2, TFAP2C, KRT7, α-hCG or β-hCG; HLA-G and GCM1. Protein levels were assayed and quantified by densitometry of each protein normalized to ACTA. Western blots show three replicate experiments for each marker for two independent rounds of differentiation. Densitometry values were used for graphs in Figures 3-5.

Supplementary Fig. S4-S5. Transcript levels of pluripotency and differentiation markers
a-k: graphs of relative transcript levels of each marker gene assayed by qRT-PCR and quantified using ΔΔCt normalized to *ACTA* in differentiation media (D) compared to the corresponding pluripotency media (P) mTeSR1 and MEF-CM. The difference between these figures and figures 2-6 is that here the E7-BAP is compared to mTeSR1 to show that there is no bias from comparison to E8. Days in culture in each differentiation medium and matching pluripotency medium are shown on the X-axis. Relative transcript levels and number of replicates for each gene are on the Y-axis. Data are presented as mean ± standard error of the mean (SEM); The difference between S4 and S5 is in S5 we have shown the expression as single dots corresponding to individual experimental replicates. *$p < 0.05$, **$p < 0.01$, ***$p < 0.001$, ****$p < 0.0001$.

Supplementary Fig. S1. – page 1

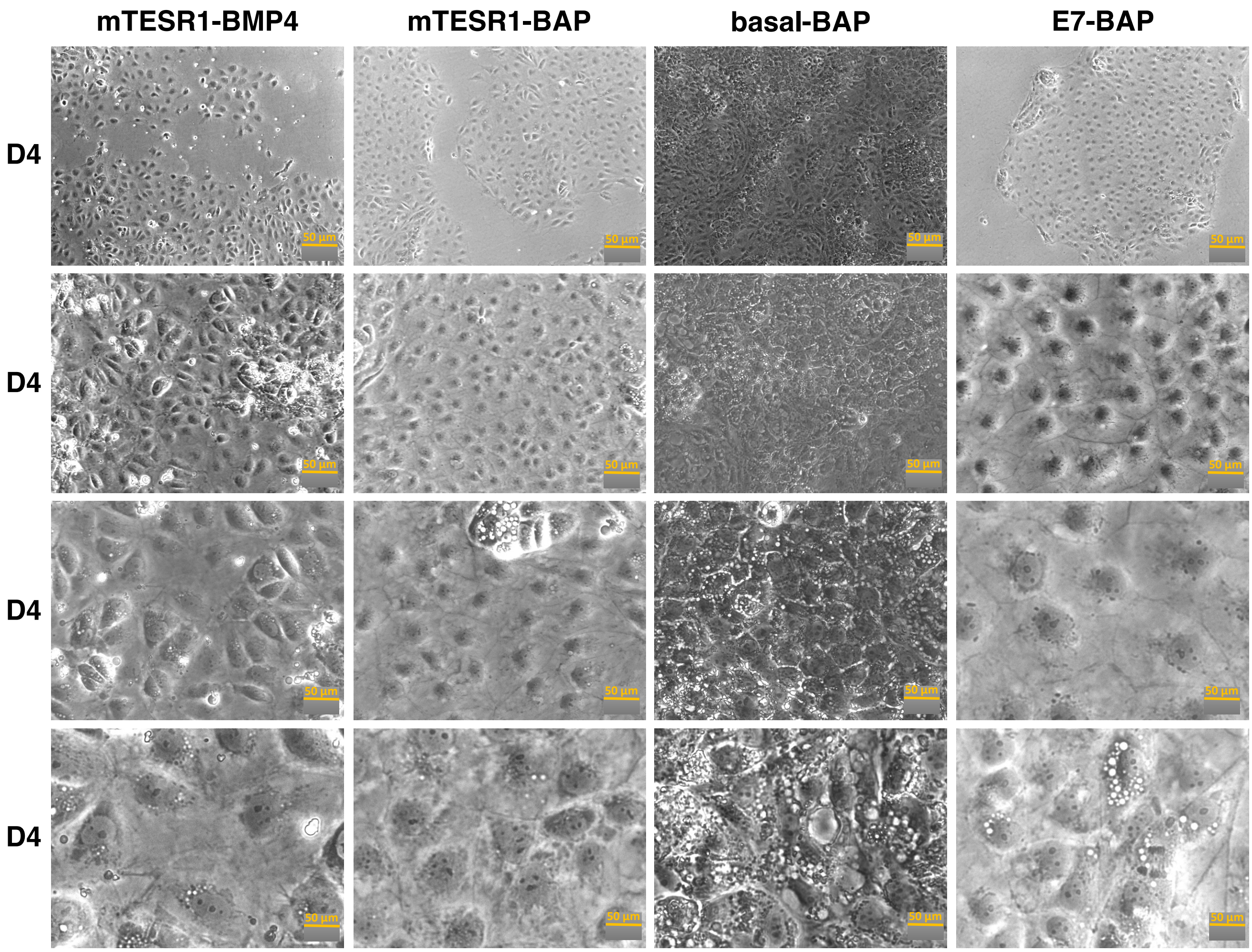

Supplementary Fig. S1. – page 2

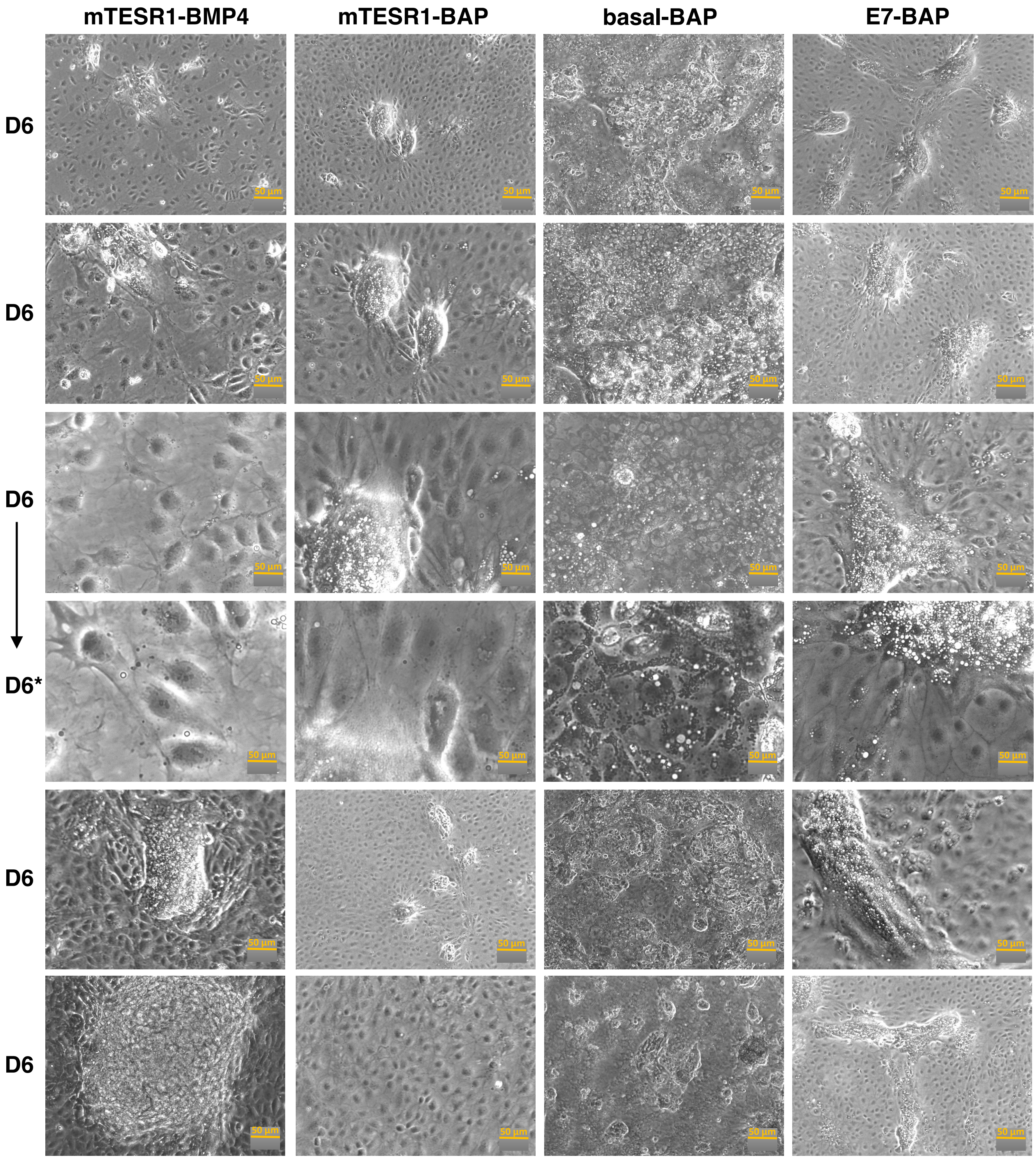

Supplementary Fig. S1. – page 3

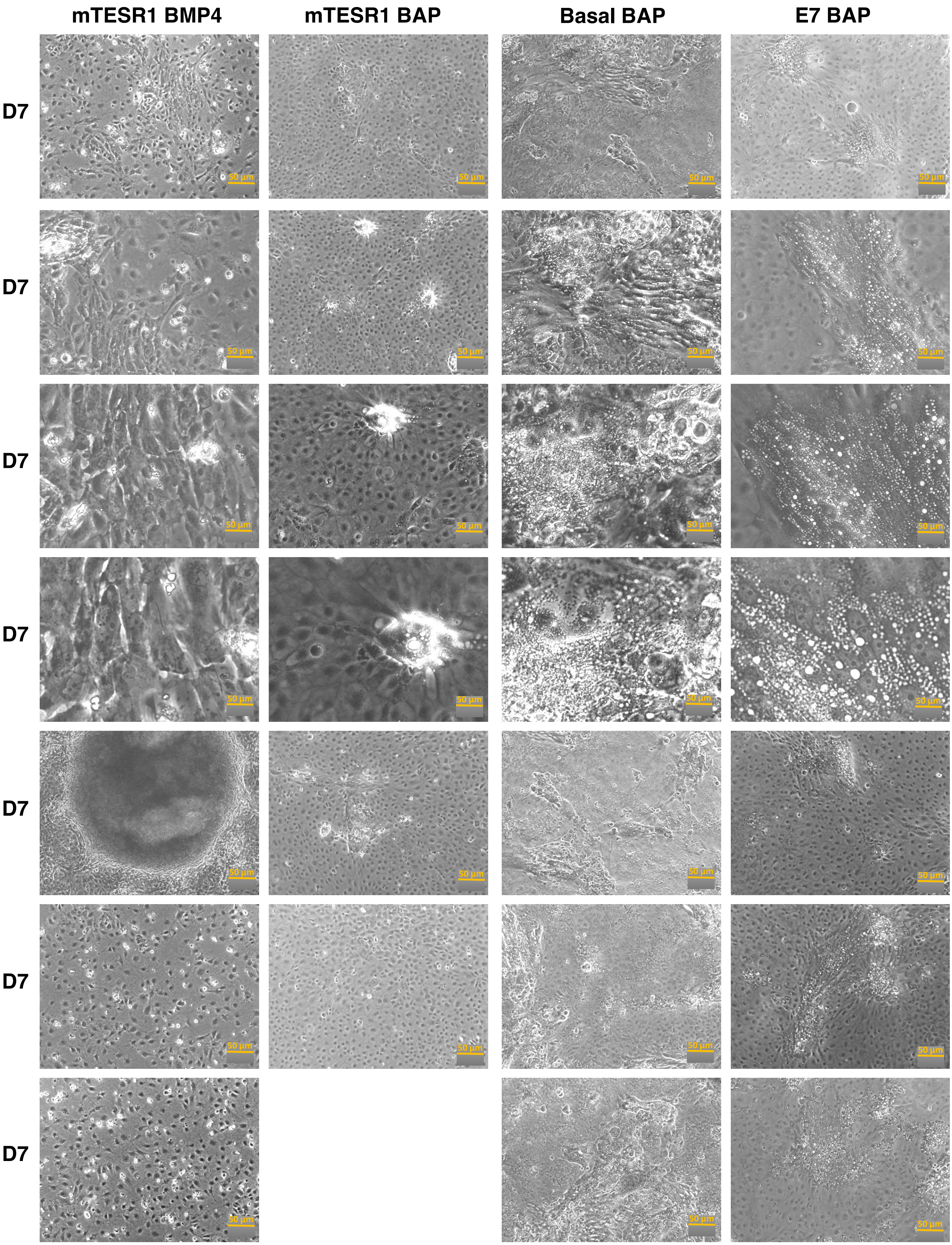

Supplementary Fig. S2

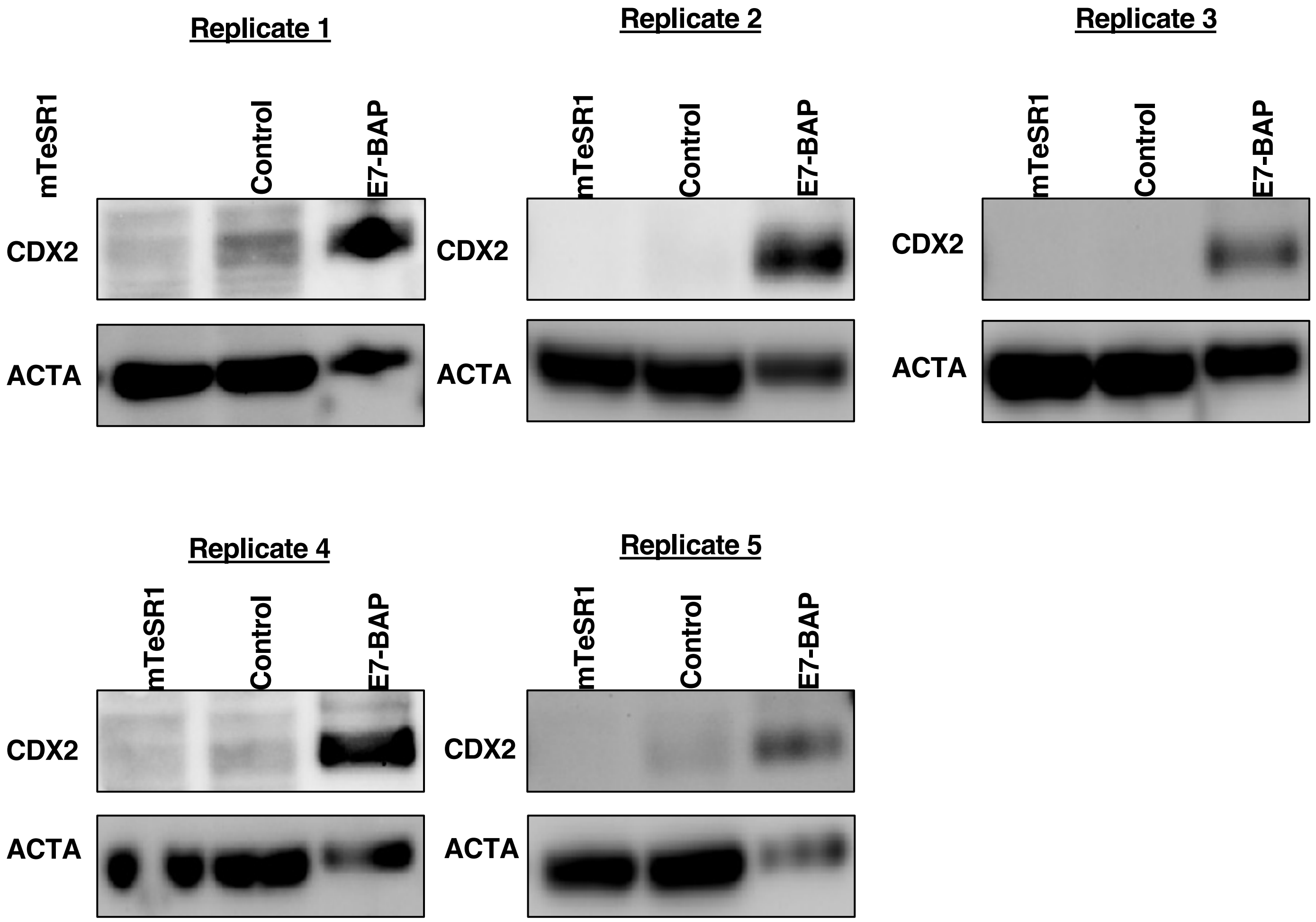

Supplementary Fig. S3

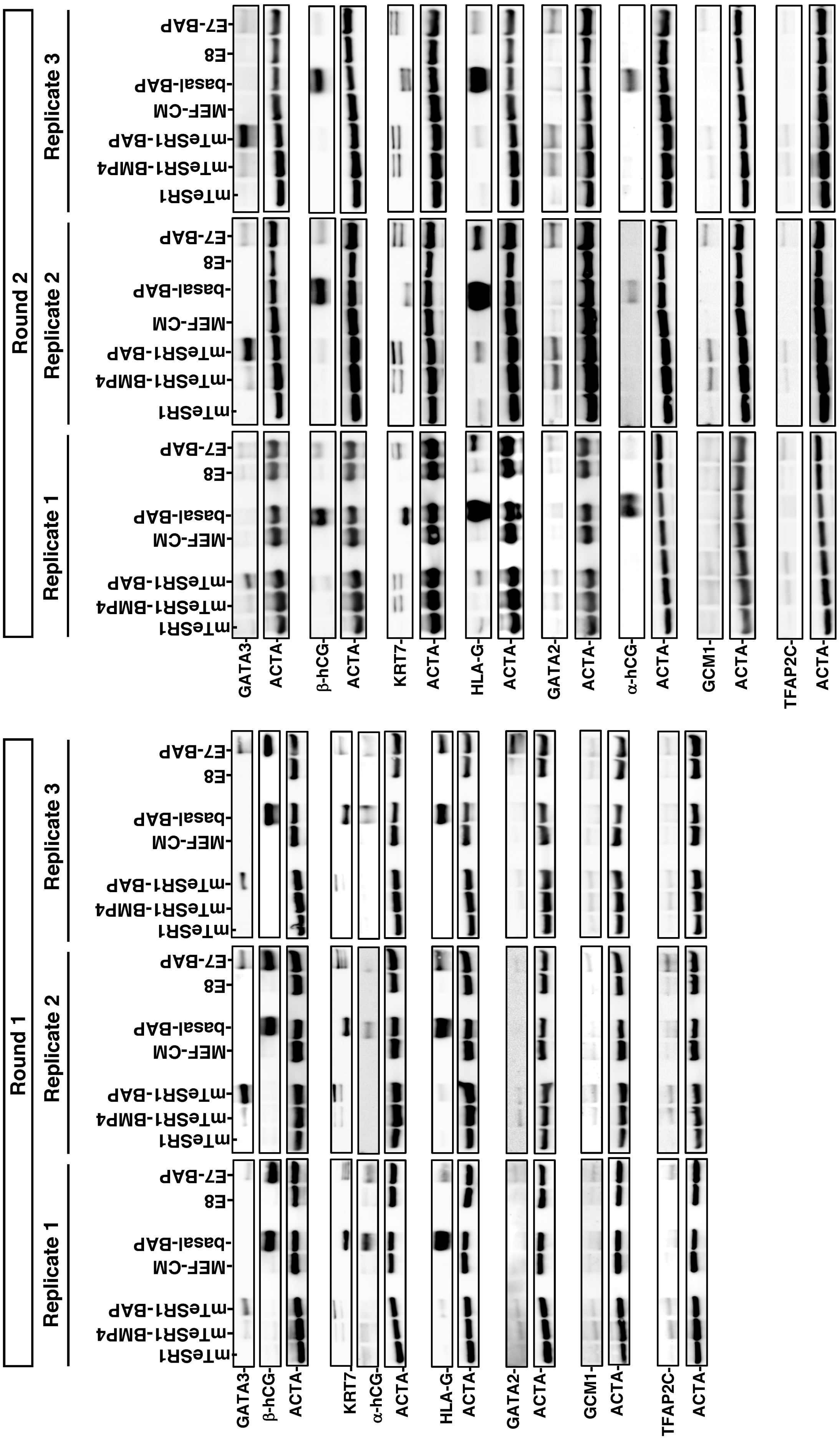

Supplementary Fig. S4

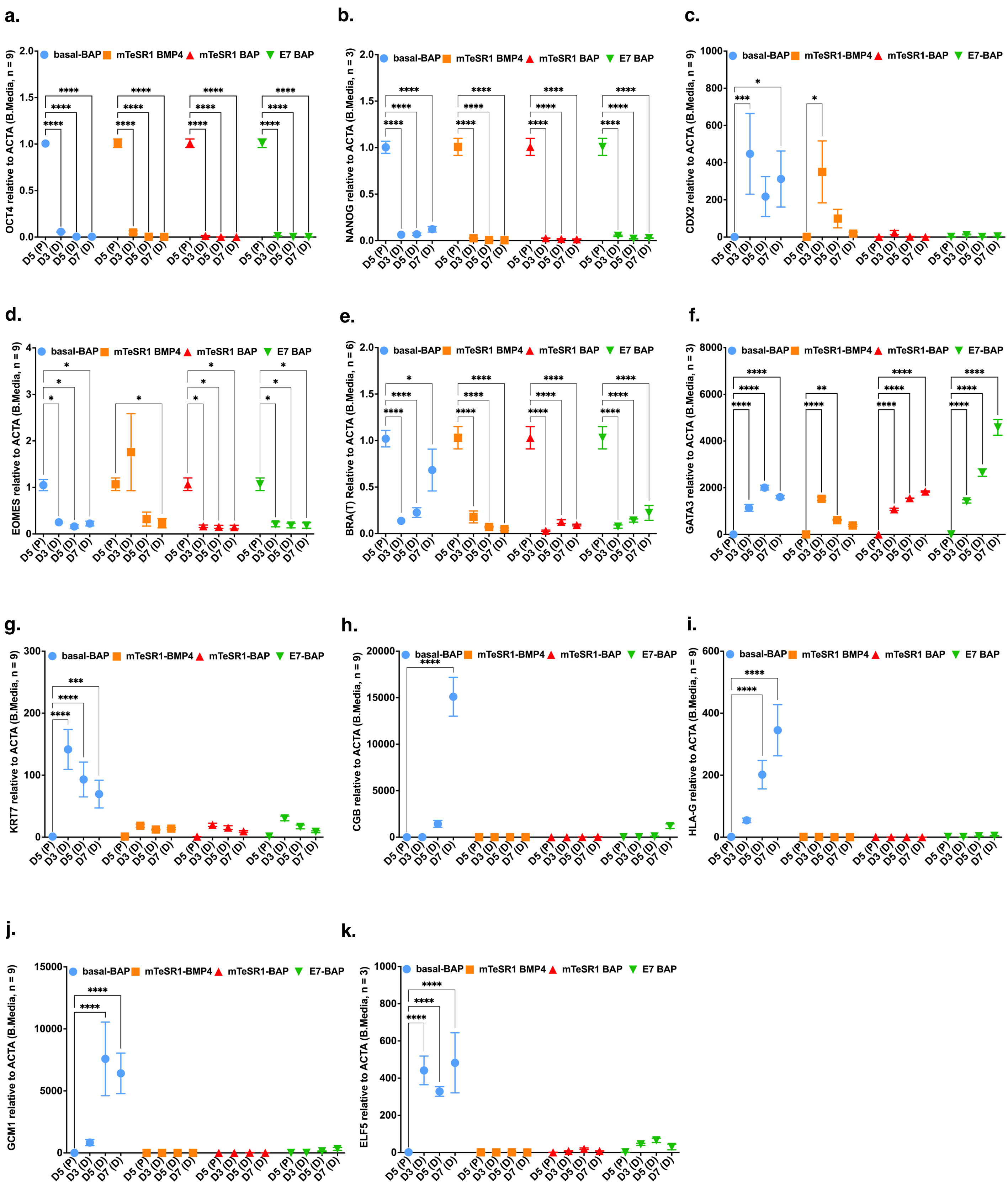

Supplementary Fig. S5

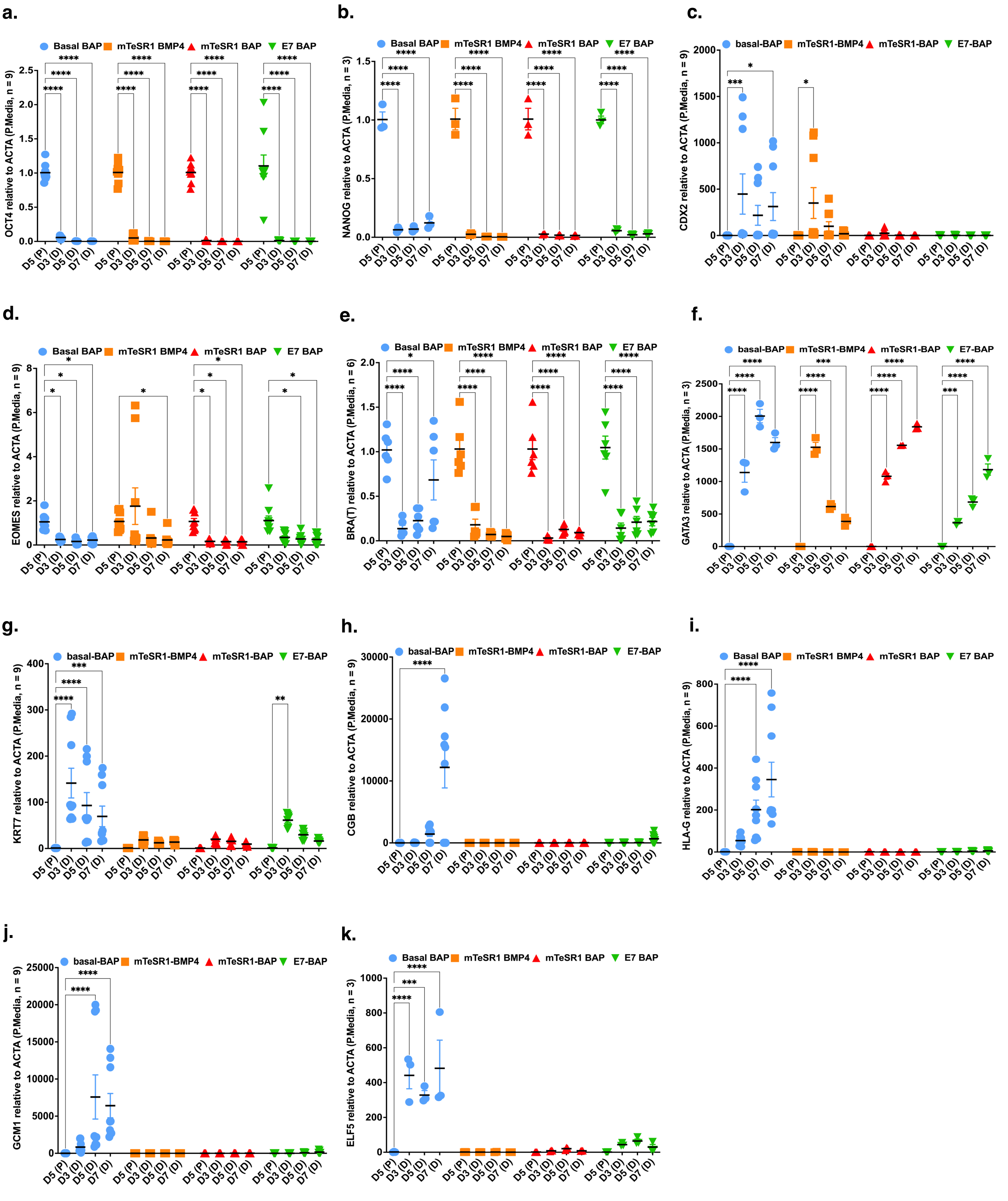

Supplement: Supplementary file 1 — Supplementary file1 (PDF 15520 KB) [file 43032_2023_1334_MOESM1_ESM.pdf]
