# Supplementary material for: Comparison of Four Protocols for In Vitro Differentiation of Human Embryonic Stem Cells into Trophoblast Lineages by BMP4 and Dual Inhibition of Activin/Nodal and FGF2 Signaling
Source: Reprod Sci. 2023 Sep 1;31(1):173–89. doi: 10.1007/s43032-023-01334-5 (PMC10784360; doi:10.1007/s43032-023-01334-5)
Supplement: Supplementary file 2 — Supplementary file2 (DOCX 28 KB) [file 43032_2023_1334_MOESM2_ESM.docx]

# Supplementary files (Table 1-4) for:

Anvar Z, Chakchouk I, Sharif M, Mahadevan S, Su L, Anikar S, Alivi F, Budi Utama A, Van den Veyver IB.

Comparison of four protocols for in vitro differentiation of human embryonic stem cells into trophoblast lineages by BMP4 and dual inhibition of Activin/Nodal and FGF2 signaling.

***In:*** *Reproductive Sciences 2022*

***Correspondence:*** Ignatia B Van den Veyver: [iveyver@bcm.edu](mailto:iveyver@bcm.edu)

# Supplementary Table. S1. Primers for qRT-PCR of mRNA

| **Gene** | **Forward primer** | **Reverse primer** |
| --- | --- | --- |
| ***ACTA*** | TCCCTGGAGAAGAGCTACGA G | TGGATGCCACAGGACTCCAT |
| ***GCM1* [6]** | TCTCTTCACCTACGCCTCTCA TC | GATCCAAACCCAAGTATGTC ATTTC |
| ***KRT7* [6]** | AGATCGCCACCTACCGCAAG | ATTCACGGCTCCCACTCCAT |
| ***CGB* [6]** | GTGCATCACCGTCAACACCA | CACATCGCGGTAGTTGCACA |
| ***CDX2/1* [6]** | TCACTACAGTCGCTACATCA  CCATC | TTAACCTGCCTCTCAGAGAG  CC |
| ***CDX2/*2 [54]** | CCCTCGGCAGCCAAGTGAAA | TCCTCCGGATGGTGATGTAG |
| ***BRA(T)* [6]** | CCTTGCTCACACCTGCAGTA GC | GGCCAACTGCATCATCTCCA |
| ***EOMES* [19]** | CGGCCTCTGTGGCTCAAA | AAGGAAACATGCGCCTGC |
| ***OCT4*** | GCCGGTTACAGAACCACACT | AGTGAGAGGCAACCTGGAGA |
| ***GATA3* [16]** | ACTCCAGCCACATGCTGACC | AGCATCGAGCAGGGCTCTAA  C |
| ***NANOG* [30]** | TGATTTGTGGGCCTGAAGAA  A. | GAGGCATCTCAGCAGAAGAC  A |
| ***ELF5* [22]** | GACGCTGAAGAAAGCAAGG  C | CCCATTCCAGAATGCCACAG |
| ***HLA-G* [31]** | GCTGCCCTGTGTGGGACTGA  GTG | GACGGAGACATCCCAGCCCC  TTT |

**Supplementary Table. S2. Primers for qRT-PCR of miRNA [20]**

| **Gene** | **RT primer** | **Forward primer** |
| --- | --- | --- |
| ***miR-103a*** | GTTGGCTCTGGTGCAGGGTCCGAGGTAT TCGCACCAGAGCCAACTCATAG | GTAGCAGCATTGTACAG GG |
| ***miR-526b- 3p*** | GTTGGCTCTGGTGCAGGGTCCGAGGTAT TCGCACCAGAGCCAACGCCTCT | GTTTGGGAAAGTGCTTCC TTTT |
| ***miR-517a*** | GTTGGCTCTGGTGCAGGGTCCGAGGTAT TCGCACCAGAGCCAACACACTC | GTTTGGATCGTGCATCCT TTTA |
| ***miR-517b*** | GTTGGCTCTGGTGCAGGGTCCGAGGTAT  TCGCACCAGAGCCAACAGACAG | GTGCCTCTAGATGGAAG  CA |
| ***miR-525-***  ***3p*** | GTTGGCTCTGGTGCAGGGTCCGAGGTAT  TCGCACCAGAGCCAACCGCTCT | GTTGAAGGCGCTTCCCTT  T |
| **Uni.**  **Reverse Primer** | GTGCAGGGTCCGAGGT |  |

**Supplementary Table. S3. Details on antibody dilutions and diluents used for each primary antibody**

| **Manufacturer** | **Antibody** | **Catalog #** | **Clone** | **Species** | **Mono/ Polyclonal** | **WB** | **IF** |
| --- | --- | --- | --- | --- | --- | --- | --- |
| **R&D Systems** | GATA3 | AF2605 | P23771 | Goat | Polyclonal | 1:1000  (5% BSA in PBST) | 1:250  (5% NGS in PBST) |
|  | α-hCG | MAB4169 | P01215 | Mouse | Monoclonal | 1:500  (5% blocker in PBST) | 1:250  (5% NGS in PBST) |
| **Abcam** | β -hCG | ab9376 | - | Rabbit | Polyclonal | 1:1000  (5% BSA in PBST) | 1:250  (5% NGS in PBST) |
| **Santa Cruz Biotechnology** | KRT7 | sc-23876 | RCK105 | Mouse | Monoclonal | 1:1000  (5% BSA in PBST) | 1:250  (5% NGS in PBST) |
|  | HLAG | sc-21799 | 4H84 | Mouse | Monoclonal | 1:1000  (5% BSA in PBST) | 1:250  (5% NGS in PBST) |
|  | GATA2 | sc-267 | CG2-96 | Mouse | Monoclonal | 1:2000  (5% BSA in PBST) | 1:250  (5% NGS in PBST) |
|  | GCM1 | sc-101173 | R-06 | Mouse | Monoclonal | 1:1000  (5% blocker in PBST) | 1:250  (5% NGS in PBST) |
|  | OCT4 | sc-5279 | C-10 | Mouse | Monoclonal |  | 1:100  (5% NGS in PBST) |
| **Cell Signaling Technology** | CDX2 | CST-3977 | _ | Rabbit | Polyclonal | 1:1000  (5% blocker in PBST) |  |
| **Developmental Hybridoma Bank, University of Iowa.** | TFAP2A | PCRP-TFAP2A - 3B5 | 3B5 |  |  |  | 1:250  (5% NGS in PBST) |
|  | TFAP2B | PCRP-TFAP2B- 2A4 | 2A4 |  |  |  | 1:250  (5% NGS in PBST) |
|  | TFAP2C | PCRP-TFAP2C- 1A7 | 1A7 |  |  | 1:2000  (5% BSA in PBST) | 1:250  (5% NGS in PBST) |
|  | CDX2 | PCRP-CDX2-1A6 | 1A6 |  |  |  | 1:250  (5% NGS in PBST) |
| **Invitrogen** | Goat anti-Mouse IgG Alexa Fluor 488 | A-11029 |  | Goat | Polyclonamal | WB | 1:1000  (5% NGS in PBST) |
| **Jackson Immuno Research** | Goat anti rabbit IgG (H+L) | 111-165-144 |  | Goat | Polyclonal |  | 1:1000  (5% NGS in PBST) |
| **Thermo Fisher Scientific** | Chicken anti-Goat IgG (H+L) | A-21469 |  | Chicken | Polyclonal |  | 1:1000  (5% NGS in PBST) |
|  | Goat anti-Rabbit IgG (H+L) Alexa Fluor™ 594 | A-11012 |  | Goat | Polyclonal |  | 1:1000  (5% NGS in PBST) |
|  | Goat anti-Mouse IgG (H+L)  Alxa Fluor™ 594 | A-11005 |  | Goat | Polyclonal |  | 1:1000  (5% NGS in PBST) |

**Supplementary Table. S4. Differences in silencing of pluripotency markers, *CGB*, *CGA*, *HLA-G*, *GCM1* transcript and protein levels and *ELF5* methylation status between the examined media**

| **Media** | **Complete silencing of pluripotency markers** | ***CGB*** | ***CGA*** | ***HLA-G*** | ***GCM1*** | ***ELF5*** |
| --- | --- | --- | --- | --- | --- | --- |
| mTeSR1-BMP4 | Yes | -  Observed protein levels on day 7 | Weak protein levels on D7 | -  - | -  Low protein levels | Promoter hypomethylation  - |
| mTeSR1-BAP | Yes | -  - | - | -  - | -  Low protein levels | Promoter hypomethylation.  - |
| E7-BAP | Yes | -  Observed protein levels on day 7 | - | -  High levels of HLA-G protein confirmed by WB | -  Low protein levels | The most Promoter hypomethylation  - |
| Basal-BAP | some cells showed possible nuclear staining at D7 | Transcript levels increased after D5 and with significantly elevated levels on D7  Highest protein levels on day 7 | Highest protein levels on D7 | Highest levels of *HLA-G* transcripts  High levels of HLA-G protein confirmed by WB and IF | Transcript levels increased gradually  Low protein levels | Promoter hypomethylation  Significant gene upregulation |
